# Extracellular Vesicle‐Transferred ATP‐Citrate Lyase Induces Monocyte Differentiation Toward Tumor‐Associated Macrophages and Fuels Hepatocellular Carcinoma Progression

**DOI:** 10.1002/advs.202521458

**Published:** 2026-04-17

**Authors:** Zhijun Liu, Haihong Lai, Qing Yang, Chaoyue Zhang, Bin Jia, Qiyi Chen, Yuling Deng, Fen Cao, Yuyu You, Zhe Li, Dongming Kuang, Bo Li

**Affiliations:** ^1^ State Key Laboratory of Multi‐organ Injury Prevention and Treatment Department of Biochemistry and Molecular Biology School of Basic Medical Sciences Southern Medical University Guangzhou China; ^2^ Guangdong Provincial Key Laboratory of Molecular Tumor Pathology Guangzhou China; ^3^ Center For Precision Medicine The First Affiliated Hospital Zhongshan School of Medicine Sun Yat‐sen University Guangzhou China; ^4^ College of Engineering and Applied Sciences Nanjing University Nanjing China; ^5^ School of Pharmaceutical Sciences Sun Yat‐sen University Guangzhou China; ^6^ School of Life Sciences Sun Yat‐sen University Guangzhou China

**Keywords:** ACLY, extracellular vesicle, hepatocellular carcinoma, macrophage, palmitoylation

## Abstract

Tumor‐associated macrophages (TAMs) arise from monocytes and represent major contributors to the immunosuppressive microenvironment of solid tumors. However, the environmental cues that govern TAM differentiation and immunosuppressive activity remain incompletely understood. Here we demonstrate that hepatocellular carcinoma (HCC) cells secrete extracellular vesicles (EVs) that are preferentially taken up by monocytes, inducing their differentiation to TAMs characterized by a distinct immune‐inhibitory signature. Mechanistically, HCC‐derived EVs encapsulate the lipogenic enzyme ATP‐citrate lyase (ACLY), promote palmitate biosynthesis in targeted monocytes, thereby enhancing the S‐palmitoylation and stability of multiple immune checkpoint proteins. To validate this, we synthesized liposomal vesicles (LVs) decorated with an EV‐marker protein CD81, which mimicked the targeting specificity of endogenous EVs for monocytes and differentiated macrophages. When loaded with ACLY proteins as interior cargo, these LVs were sufficient to induce immunosuppressive TAMs and promote HCC progression. Conversely, CD81‐decorated LVs encapsulating the ACLY inhibitor SB204990 markedly reduced the TAM‐mediated immunosuppressive activity, leading to restrained HCC progression. Importantly, we further demonstrated that targeting EV‐transferred, TAM‐specific ACLY represents a promising strategy to enhance immunotherapeutic efficacy without notable side effects, particularly when combined with anti‐PD‐1/PD‐L1 antibodies for HCC treatment.

## Graphical abstract

Hepatocellular carcinoma (HCC)‐derived extracellular vesicles (EVs) enrich the metabolic enzyme ATP‐citrate lyase (ACLY). EV‐transferred ACLY enhances palmitate biosynthesis, increases the S‐palmitoylation and stability of multiple immune checkpoint proteins, augments the cellular immunosuppressive activity, and ultimately accelerates the malignant progression of HCC. Furthermore, biomimetic nanovesicles delivering a preclinical ACLY inhibitor in combination with anti‐PD‐1 therapy present a promising combination strategy for HCC.

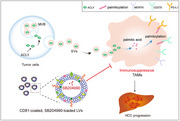

**Abbreviations**
ACLY: ATP‐citrate lyase
DEN: diethylnitrosamine
EV: extracellular vehicle
HCC: hepatocellular carcinoma
KC: Kupffer cells
LV: liposomal vesicle
MoMF: monocyte‐derived macrophages
SCLV: SB204990‐loaded, CD81‐coated LV
TAMs: Tumor‐associated macrophages

## 1 Introduction

Immunotherapy has emerged as a breakthrough in cancer treatment primarily focusing on T cell activation, given that cytotoxic T lymphocytes are central executors to eliminate tumor cells [1]. Despite the wide and growing application of anti‐PD‐1/PD‐L1 antibodies in clinics, these immunotherapies exhibited limited efficacy due in large part to the immunosuppressive activity of myeloid cells in the tumor microenvironment (TME) [1]. Multiple subpopulations of myeloid cells, especially tumor‐associated macrophages (TAMs) differentiated from monocytes, play a critical role in suppressing the anti‐tumor immune response in solid tumors [2]. TAMs hinder immunotherapeutic efficacy largely through immune checkpoint proteins, such as the B7 family members (CD274/PD‐L1, CD276/B7‐H3) [3], MER proto‐oncogene tyrosine kinase (MERTK) [4], the “don't eat me” signal effectors (SIRPα‐CD47, SIGLEC‐7/9/10) [5], etc. Unfortunately, recent strategies targeting the SIRPα‐CD47 axis have been halted given unfavorable side effects in treated patients [2]. Therefore, a deeper understanding of the molecular mechanism that drives TAM differentiation toward protumorigenic status is critical for identifying new strategies to improve the safety and efficacy of cancer immunotherapies.

TME is composed of matrix proteins, cytokines, metabolites, etc. [6]. These factors work in concert to promote the differentiation of monocytes to protumorigenic TAMs including M2‐like macrophages [7]. Whereas effector proteins including arginase‐1 (ARG1) [8], vascular endothelial growth factor (VEGF) [9, 10], and various cytokines (IL10, TGF‐β, etc.) [11] are essential in shaping monocytes toward the immunosuppressive phenotype, emerging evidence highlights the importance of deregulated metabolic status in macrophage education [12, 13, 14]. For instance, 25‐hydroxycholesterol in lysosomes competes with cholesterol for binding to G protein‐coupled receptor 155 (GPR155), inhibiting mammalian target of rapamycin complex 1 (mTORC1)A and activating AMP‐activated protein kinase alpha (AMPKα), thereby promoting TAM differentiation [15]. Moreover, lactate and nucleotides derived from efferocytosis regulate macrophage fate decision and anti‐immune responses [16]. In addition to these metabolites, macrophage metabolism and activities are also modulated by tumor‐derived extracellular vesicles (EVs), which are lipid bilayer‐enclosed vesicles consistently released from tumor cells and fuse with neighboring cells in the TME [17, 18, 19]. Specifically, EVs contribute to tumor immune evasion by transferring bioactive molecules that suppress the function of various immune cells, such as T cells and natural killer (NK) cells, and by promoting an immunosuppressive tumor microenvironment [20]. For instance, tumor derived EVs carry immunosuppressive factors like PD‐L1, TGF‐β, and specific microRNAs, which can induce T cell exhaustion and dysfunction, thereby facilitating tumor progression and metastasis [20, 21]. Conversely, EVs may also carry tumor antigens or immunostimulatory signals that contribute to the activation and regulation of immune responses, thereby potentially enhancing the body's capacity to combat cancer [22]. This dual role highlights the complexity of EVs in tumor immunity and underscores their potential as both therapeutic targets and vehicles in cancer immunotherapy.

EVs can be classified into microvesicles and exosomes, both of which contain bioactive cargo including miRNAs and proteins derived from source cells [23, 24]. Tumor‐derived EVs are increasingly recognized for their role in macrophage education in various cancer types, primarily by creating immunosuppressive niche in the TME and metastatic sites [19]. For instance, tumoral EVs can target and reprogram Kupffer cells (KCs), the resident tissue macrophages of the liver, leading to accelerated hepatic steatosis and progression of hepatocellular carcinoma (HCC) [18]. Fatty liver‐derived EVs elevated M2 macrophage infiltration and promote the growth of colorectal cancer liver metastasis [25]. However, the specific EV components that govern macrophage fate and function in the TME remain incompletely explored.

Given the compromised efficacy of immunotherapies in treating HCC [26], we employed HCC as the tumor model to elucidate molecular mechanisms underlying TAM differentiation in the TME. We discovered that ATP‐citrate lyase (ACLY), which is a key enzyme for fatty acid biosynthesis, is packaged into HCC‐derived EVs. ACLY promotes palmitate biosynthesis, enhances the S‐palmitoylation and stability of multiple immune checkpoint proteins in EV‐targeted TAMs, and ultimately accelerates HCC progression. To further establish these findings, we constructed biomimetic liposomal vesicles (LVs) resembling natural EVs, with designed capacity of loading proteins onto the surface and/or inside of LVs. We observed that EVs or LVs carrying ACLY significantly promote TAM differentiation and HCC progression. In contrast, LVs delivering the ACLY inhibitor SB204990 to recipient monocytes/macrophages markedly inhibit HCC progression. Moreover, administration of SB204990‐containing LVs can enhance immunotherapeutic efficacy by inducing a pronounced synergistic effect with anti‐PD‐1/PD‐L1 antibodies.

## 2 Results

### 2.1 Monocyte‐Derived Macrophages Confer Immunosuppression in HCC

**FIGURE 1:**
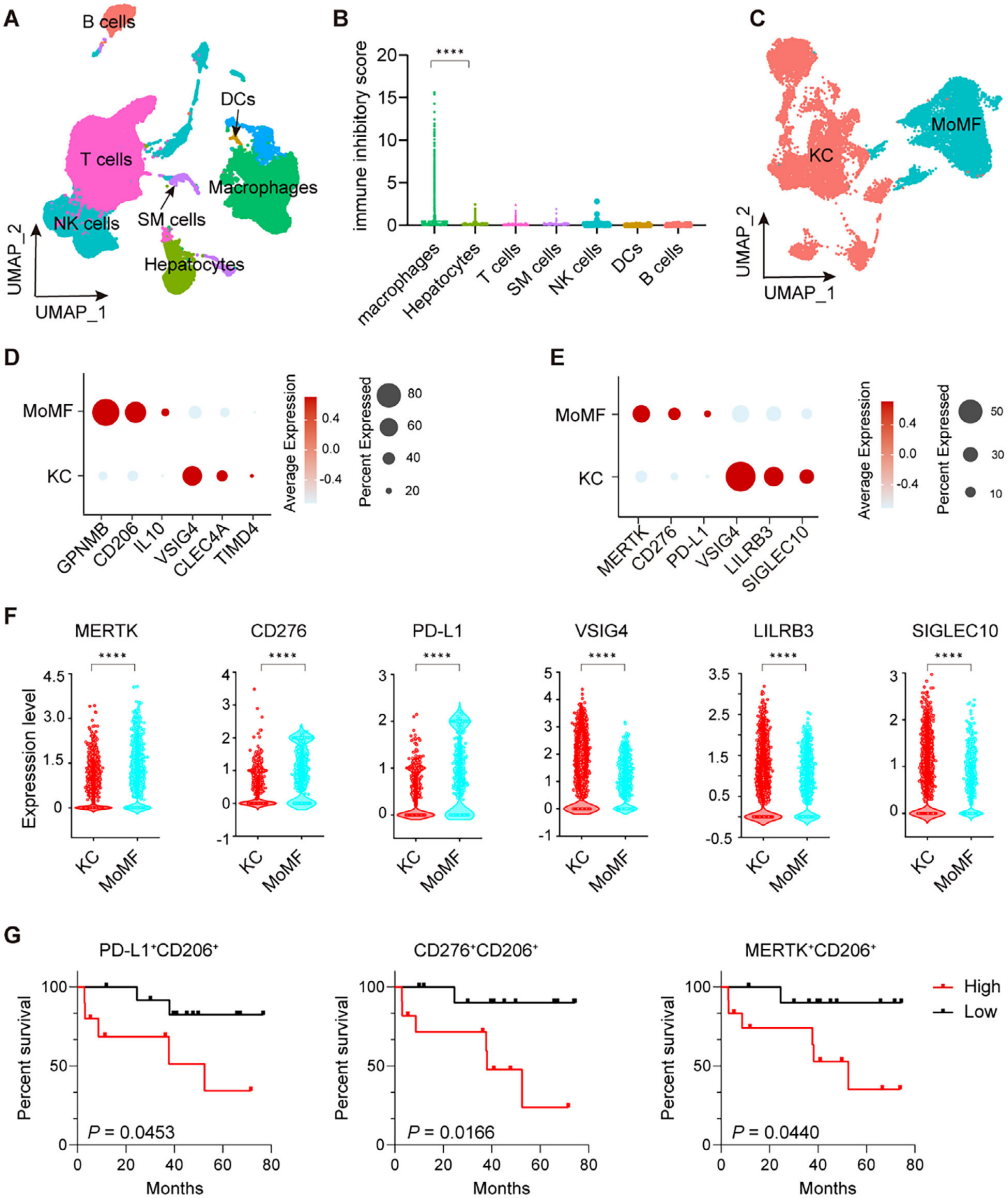
Macrophages in HCC tissues exhibit strong immunosuppressive activity. (A) Uniform Manifold Approximation and Projection (UMAP) visualization of HCC landscape based on single‐cell RNA‐sequencing (scRNA‐seq) data from public GEO datasets (GSE166635, GSE140228, and GSE138709). SM cells: smooth muscle cells. DCs, dendritic cells. NK cells, natural killer cells. (B) Immune inhibitory scores for indicated cell types in HCC tissues based on scRNA‐seq data from GEO datasets (GSE166635, GSE140228, and GSE138709). (C) UMAP visualization of monocyte‐derived macrophages (MoMFs) and Kupffer cells (KCs) of the macrophage subpopulation in HCC tissues based on scRNA‐seq data from GEO datasets (GSE166635, GSE140228, and GSE138709). (D) Dot plot showing the scaled expression of lineage‐specific marker genes in two macrophage subpopulations based on scRNA‐seq data from GEO datasets (GSE166635, GSE140228, and GSE138709). Dot color intensity indicates scaled average expression. (E,F) Dot plot presentation (E) and relative expression (F) of indicated immune‐inhibitory gene and KCs based on scRNA‐seq data from GEO datasets (GSE166635, GSE140228, and GSE138709). (G) Kaplan–Meier overall survival analysis of MERTK^+^CD206^+^, PD‐L1^+^CD206^+^, and CD276^+^CD206^+^ cells in human HCC tissue sections of a local cohort (*n* = 23, high: *n* = 12, low: *n* = 11). Data in different patient groups were statistically compared by log‐rank *t* test. Data are presented as mean ± standard deviation (SD). ****p < 0.0001 (unpaired two‐tailed Student's *t*‐test).

Immunosuppressive myeloid cells significantly undermine the efficacy of PD‐1/PD‐L1 therapeutics [1], yet the precise mechanisms governing this effect remain far from well understood. To explore this, we analyzed single‐cell transcriptome profiles of HCC tissues obtained from publicly available databases. The single‐cell Uniform Manifold Approximation and Projection (UMAP) visualization revealed distinct clusters of intratumoral cells, including hepatocytes, T cells, NK cells, macrophages, B cells, dendritic cells (DCs), and smooth muscle (SM) cells, indicating the complex cellular landscape within HCC (Figure 1A). Next, we examined the expression of reported immune‐inhibitory markers across different immune cell populations, and observed that the macrophage subset exhibited highest inhibitory scores (Figure 1B). These results suggested that macrophages, compared with other immune cells, play a prominent role in the immunosuppressive environment of HCC. This aligns with previous studies in colorectal cancer, reinforcing the critical role of macrophages in promoting immune evasion across different solid tumor types [27, 28].

The hepatic macrophage population is largely composed of KCs and monocyte‐derived macrophages (MoMFs) [29], which were naturally distinguished by UMAP analysis (Figure 1C). Kupffer cells are resident liver macrophages originating from embryonic yolk sac and hematopoietic stem cells, whereas MoMFs differentiate from circulating monocytes in the peripheral blood [30]. Further UMAP analysis confirmed specific markers for these two subpopulations based on previous studies: KCs were characterized by the expression of V‐Set And Immunoglobulin Domain Containing 4 (VSIG4), C‐Type Lectin Domain Family 4 Member A (CLEC4A), and T Cell Immunoglobulin And Mucin Domain Containing 4 (TIMD4), whereas MoMFs were defined by the expression of CD206, Glycoprotein Non‐Metastatic Melanoma Protein B (GPNMB), and IL10 [29, 31, 32, 33] (Figure 1D and Figure S1A). Both macrophage subsets are implicated in the progression of multiple liver diseases through distinct yet partially overlapping mechanisms [29, 31].

Compared with KCs, we found that MoMFs expressed significantly higher levels of immune checkpoint proteins including MERTK, CD276, and PD‐L1, suggesting that MoMFs are highly equipped to suppress the anti‐tumor immune responses in HCC (Figure 1E,F and Figure S1B). These three checkpoint molecules are known to be induced by tumor cells and inhibit the activation of cytotoxic immune cells in the TME, whereas KC‐expressing VSIG4, LILRB3, and SIGLEC10 are constitutively‐expressed immune receptors that function primarily in maintaining innate immune homeostasis [3, 4, 5]. Our findings were further corroborated by transcriptome analysis of The Cancer Genome Atlas (TCGA) HCC dataset and immunohistochemical studies of a local HCC cohort, revealing that the abundance of PD‐L1^+^CD206^+^, MERTK^+^CD206^+^, and CD276^+^CD206^+^ subpopulations in HCC tissues were associated with poor patient prognosis (Figure 1G and Figure S1C,D). These results collectively suggested that CD206^+^ cells are the macrophage subset predominantly involved in tumor‐associated immune escape in HCC.

### 2.2 HCC‐Derived Extracellular Vesicles Promote Monocyte Differentiation toward Immunosuppressive Macrophages

**FIGURE 2:**
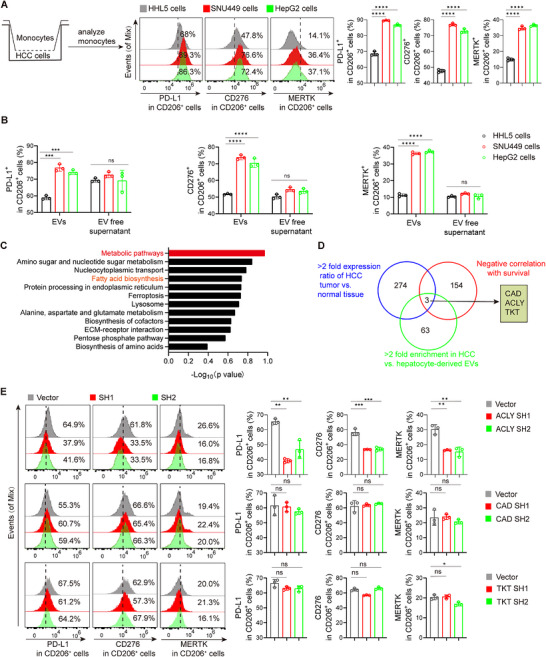
HCC‐secreted EVs drive monocyte differentiation to TAMs through ACLY. (A) Experimental design of a coculture system of HCC cells and monocytes (left), plus representative histograms (middle) and quantification (right) of immunosuppressive markers in cocultured monocytes (*n* = 3). (B) Representative quantification of flow cytometry analysis in monocytes incubated with 5 µg EVs or EV‐free supernatant secreted by hepatocyte cell line HHL5, HCC cell line HepG2 or SNU449 for 4 days (*n* = 3). (C) After quantitative proteomic analysis of EV‐containing proteins, pathway enrichment analysis was performed on identified proteins exhibiting more than twofold enrichment in HepG2 cell‐derived EVs compared to those from HHL5 cells. (D) Venn diagram showing the overlap between metabolic genes with more than twofold upregulation in The Cancer Genome Atlas (TCGA)‐collected HCC tissues compared to normal adjacent tissues, metabolic genes negatively correlated with HCC patient survival, and metabolic enzymes enriched (>two fold) at the protein level in HCC‐derived EVs. (E) Representative histograms (left) and quantifications (right) of indicated markers in monocytes incubated with 5 µg EVs secreted by HepG2 cells lentivirally transfected with vector control or two independent shRNAs respectively depleting ACLY, CAD, or TKT (*n* = 3). Data are presented as mean ± SD. ns, not significant. **p < 0.01, ***p < 0.001, ****p < 0.0001 (one‐way ANOVA with Tukey's Honestly Significant Difference (HSD) test).

Next, we asked whether specific factors derived from HCC cells were responsible for driving the differentiation of monocytes to immunosuppressive MoMFs. To examine this, we cocultured human monocytes purified from primary peripheral blood mononuclear cells (PBMCs) with two HCC cell lines SNU449 and HepG2, or the nontransformed hepatocyte cell line HHL5 seeded in Boyden chambers. Intriguingly, PBMC‐containing monocytes cocultured with HCC cells exhibited a clear immunosuppressive phenotype, marked by the upregulation of MERTK, CD276, and PD‐L1 in the differentiated CD206^+^ population (Figure 2A). In contrast, monocytes cocultured with nontransformed hepatocytes displayed attenuated expression of these immune checkpoint proteins (Figure 2A), indicating that HCC cells promote the differentiation of monocytes into immunosuppressive macrophages likely through the secretion of specific factors, yet not through direct cell–cell contact.

Whereas chemokines and cytokines have long been recognized as important mediators of monocyte differentiation, EVs recently emerged as novel players in this process [23]. To determine whether HCC‐derived EVs contribute to the immunosuppressive transformation of monocytes, we isolated EVs and EV‐free supernatants from HCC and hepatocyte cultures, and respectively incubated them with primary monocytes. To obtain EVs with high purity, we isolated EVs referring to the Minimal Information for Studies of Extracellular Vesicles (MISEV) guidelines [34, 35, 36]. The size and morphology of purified EVs were evaluated by transmission electron microscopy (TEM) and nanoparticle tracking analysis (NTA) (Figure S2A,B). EV purity was confirmed by immunoblot analysis of specific EV markers (CD81, CD9, and TSG101). Importantly, we excluded potential contamination by ApoB‐containing lipoproteins, which can resemble EVs by size and are abundantly secreted by HCC cells. (Figure S2C).

As a result, EV‐free supernatants regardless of their origin had little effect on the immunosuppressive phenotypes associated with incubated monocytes (Figure 2B and Figure S2D). Conversely, EVs secreted from HCC cells rather than hepatocytes are largely responsible for monocyte‐to‐macrophage differentiation and immunosuppression (Figure 2B and Figure S2D,E). These results were consistent with our observation that compared with other immune cell populations, EVs collected from HCC cells specifically targeted monocytes/macrophages in the circulation and liver, as demonstrated by both in vitro and in vivo experiments using DiR fluorophore‐labeled EVs (Figure S2F–I).

We then sought to identify the specific components within HCC‐derived EVs that are responsible for inducing these phenotypic shifts. Electroporation of the nucleic acid components of HCC‐derived EVs into monocytes failed to mimic the effects of EV incubation, suggesting that the EV protein components were likely involved (Figure S3A). Therefore, we employed quantitative proteomic analysis to compare EVs secreted from HCC cells (HepG2) and hepatocytes (HHL5), and observed significant alterations of metabolic enzymes in HCC‐derived EVs, in particular those orchestrating fatty acid biosynthesis (Figure 2C). Among all the proteins upregulated in HCC‐derived EVs, CAD (carbamoyl phosphate synthetase) [37], TKT (transketolase) [38], and ACLY [39] were also upregulated in HCC tumor tissues and correlated with worse patient prognosis (Figure 2D and Figure S3B,C). To determine whether these proteins drive the differentiation of monocytes into immunosuppressive macrophages, we depleted each of them in HCC cells and respectively supplemented primary monocytes with derived EVs. Remarkably, only EVs carrying replete ACLY were able to confer the immunosuppressive phenotype, demonstrating that ACLY is the critical protein cargo transferred by HCC‐derived EVs to induce TAM differentiation (Figure 2E and Figures S3D–S4C). ACLY is a key enzyme converting citrate to acetyl‐CoA for fatty acid biosynthesis [40]. We therefore speculated that HCC‐derived EVs may induce immunosuppression by reprogramming fatty acid metabolism in recipient monocytic lineages, leading to accelerated tumor progression.

### 2.3 ACLY Protein Transferred by HCC‐Derived EVs Promotes Tumor Growth In Vivo

**FIGURE 3:**
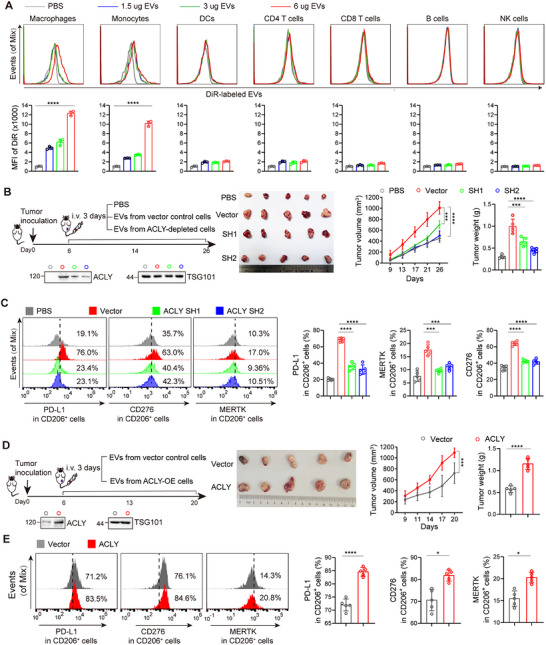
EV‐containing ACLY promotes HCC tumor growth by increasing the abundance of immunosuppressive TAMs. (A) Uptake of DiR‐labeled EVs by immune cells from mouse liver were quantified by flow cytometry after intravenous injection (i.v.) of phosphate buffered saline (PBS) control or EVs for 3 h. Top: representative histograms. Bottom: quantification of fluorescence intensity of DiR‐positive immune cells incubated with PBS control or various amounts of EVs (*n* = 3). (B) Flowchart illustrating the experimental design (left), images and growth (middle), and weights (right) of subcutaneous tumors i.v. treated with PBS or 10 µg EVs derived from Hepa1‐6 cells transfected with vector control or shRNAs depleting ACLY (*n* = 5). Immunoblots showing the protein levels of ACLY were provided on the right. Representative of *n* = 3 independent experiments. (C) Flow cytometry analysis of immunosuppressive TAMs in subcutaneous tumors as indicated in (B). Left: representative histograms. Right: quantification of histograms (*n* = 5). (D) Experimental design (left), images and growth (middle), and weights (right) of subcutaneous tumors treated with intravenous injection of PBS or 5 µg EVs derived from Hepa1‐6 cells transfected with vector control or overexpressing ACLY (ACLY OE) (*n* = 5). Immunoblots showing the protein levels of ACLY were provided. Representative of *n* = 3 independent experiments. (E) Flow cytometry analysis of immunosuppressive TAMs in subcutaneous tumors as indicated in (D). Left: representative histograms. Right: quantification of histograms (*n* = 5). (A–C) one‐way ANOVA with Tukey's HSD test. (D,E) Two‐tailed, unpaired Student's *t*‐test. Data are presented as mean ± SD. *p < 0.05, ***p < 0.001, ****p < 0.0001.

To prove the above hypothesis, we sought to determine whether the effect of HCC‐derived EVs can be extended to in vivo tumor models. Therefore, we labeled HCC‐derived EVs with the DiR fluorophore and intravenously injected them into HCC tumor‐bearing mice to evaluate their uptake efficiency. Consistent with our previous data in blood samples (Figure S2E–G), these EVs can also efficiently target monocytic lineages in HCC tissues, with minimal uptake by other immune cell populations (Figure 3A). To further examine whether the EV‐mediated crosstalk between HCC cells and TAMs contributes to tumor progression, we established an immunocompetent HCC model in C57BL/6J mice using the isogenic Hepa1‐6 cell line. 6 days after tumor inoculation, mice were intravenously injected with EVs collected from Hepa1‐6 cells expressing the vector control, or ACLY‐depleted Hepa1‐6 cells expressing two independent shRNAs targeting ACLY (ACLY SH1 and SH2). Notably, mice injected with EVs from ACLY‐depleted Hepa1‐6 cells exhibited markedly reduced HCC tumor growth compared to those receiving vector control EVs, which was accompanied by a paralleled decrease in the number of immunosuppressive TAMs (Figure 3B,C). In contrast, overexpression of ACLY in Hepa1‐6 cells led to the production of EVs that markedly enhanced HCC tumor growth and increased the abundance of immunosuppressive TAMs, even with lower amounts of injected EVs (Figure 3D,E). Similar results were observed in tumor‐bearing mice receiving EVs from ACLY‑depleted SNU449 or HepG2 cells (Figure S5A‐E). These findings demonstrated that EV‐containing ACLY facilitates HCC progression by promoting tumoral accumulation of immunosuppressive TAMs.

### 2.4 ACLY‐Enriched EVs Drive TAM Polarization by Enhancing Palmitate Biosynthesis

**FIGURE 4:**
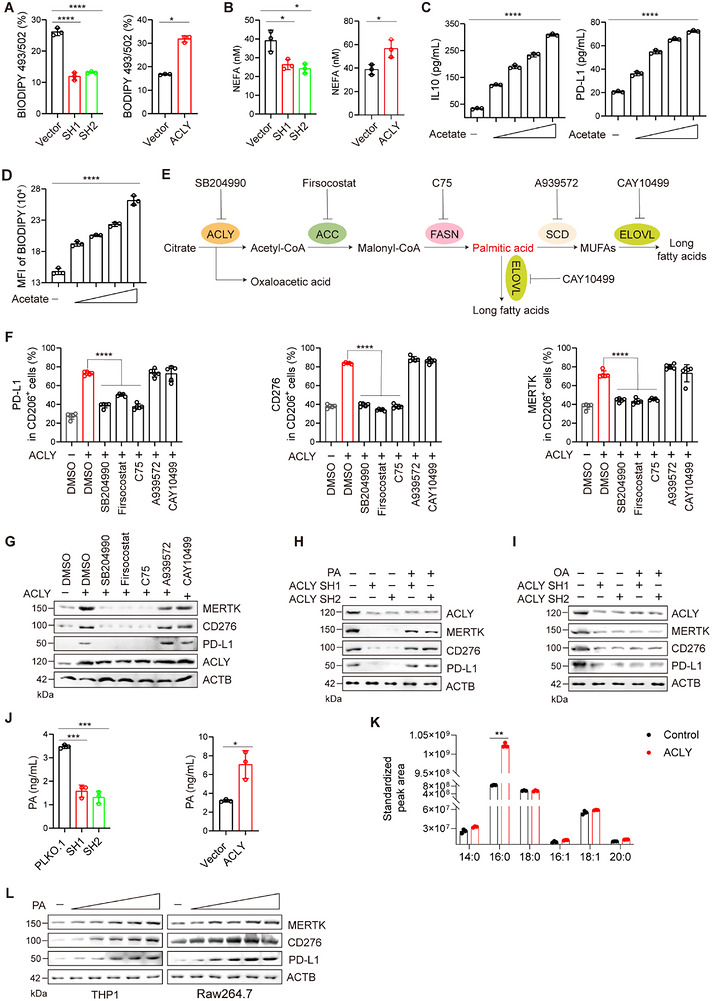
ACLY‐encapsulated EVs drive TAM polarization by producing palmitate. (A) Quantification of the lipophilic fluorescent probe BODIPY 493/503 staining in monocytes incubated with 5 µg EVs secreted from HepG2 cells with or without ACLY depletion or overexpression for 4 days (*n* = 3). (B) The nonesterified fatty acid (NEFA) level in monocytes incubated with 5 µg EVs secreted from HepG2 cells with or without ACLY depletion or overexpression for 4 days (*n* = 3). Left, one‐way ANOVA with Tukey's HSD test. Right, Two‐tailed unpaired Student's *t*‐test. (C) The enzyme‐linked immunosorbent assay (ELISA)‐determined IL10 and PD‐L1 levels in monocytes incubated without or with increasing doses of acetate (3–24 mm) for 2 days, in the presence of 5 µg EVs secreted from HepG2 cells with ACLY depletion (*n* = 3). (D) Quantification of BODIPY 493/503 staining in monocytes cultured without or with increasing doses of acetate (3–24 mm) for 2 days, in the presence of 5 µg EVs secreted from HepG2 cells with ACLY depletion (*n* = 3). (E) Schematic diagram of *de novo* fatty acid biosynthesis and indicated inhibitors. ACLY, ATP citrate lyase. ACC, acetyl‐CoA carboxylase. FASN, fatty acid synthase. SCD, stearoyl‐CoA desaturase. ELOVL, elongation of very long chain fatty acids protein. The administrated doses of inhibitors are: 10 µm SB204990, 10 µm C75, 10 nm Firsocostat, 50 nm A939572, 100 nm CAY10499. (F) Flow cytometry analysis of immunosuppressive markers in THP1 cells incubated with 5 µg of EVs secreted by HepG2 cells lentivirally transfected with vector control or ACLY for 2 days, followed by treatment with different inhibitors for another 2 days (*n* = 5). (G) Protein levels of indicated immunosuppressive markers in THP1 cells incubated with 5 µg of EVs secreted by HepG2 cells lentivirally transfected with vector control or ACLY for 2 days, followed by treatment with different inhibitors for another 2 days. Representative of *n* = 3 independent experiments. (H,I) Levels of indicated proteins in THP1 cells incubated with 5 µg EVs derived from HepG2 cells with or without ACLY depletion for 4 days, followed by treatment with 0.5 mm palmitic acid (PA) (H) or oleic acid (OA) (I) for 8 h. Representative of *n* = 3 independent experiments. (J) Lysate PA levels in monocytes incubated with 5 µg EVs secreted from HepG2 cells with or without ACLY depletion or overexpression for 4 days (*n* = 3). Left, one‐way ANOVA with Tukey's HSD test. Right, Two‐tailed unpaired Student's *t*‐test. (K) Metabolite levels in monocytes incubated with 5 µg EVs secreted from HepG2 cells with or without ACLY overexpression for 4 days by metabolomic profiling (*n* = 3). Altered fatty acid species included the saturated fatty acids myristic acid (14:0), palmitic acid (16:0), stearic acid (18:0), and arachidic acid (20:0), as well as the monounsaturated fatty acids palmitoleic acid (16:1) and oleic acid (18:1). (L) Protein levels of indicated immunosuppressive markers in THP1 (left) and Raw264.7 (right) cells treated without or with increasing doses of PA (50–500 µm) for 8 h. Representative of *n* = 3 independent experiments. (A,C,D,F) one‐way ANOVA with Tukey's HSD test. (K) Two‐tailed, unpaired Student's *t*‐test. Data are presented as mean ± SD. *p < 0.05, ***p < 0.001, ****p < 0.0001.

To elucidate the molecular mechanisms underlying EV‐mediated TAM differentiation, we manipulated ACLY levels directly in predifferentiated monocytes. Consistent with our earlier findings, ACLY attenuation in monocytes significantly inhibited their differentiation into immunosuppressive TAMs, whereas ACLY overexpression led to the opposite effects (Figure S6A,B). These results support the role of ACLY as a direct regulator of monocyte differentiation, presumably because HCC‐derived EVs transfer ACLY protein to recipient monocytes and induce their metabolic programming. ACLY plays a critical role in generating acetyl‐CoA, which is required for fatty acid biosynthesis in the cytosol and histone acetylation in the nucleus [39, 41, 42]. Interestingly, we observed little changes in several histone H3 and H4 acetylation sites in response to EV transferred‐ACLY (Figure S6C). On the contrary, cellular levels of neutral lipids and nonesterified fatty acids (NEFAs) were significantly enhanced when monocytes were exposed to ACLY‐containing EVs (Figure 4A,B), and treating these monocytes with SB204990, a specific ACLY inhibitor [39], markedly suppressed their lipid accumulation and immunosuppressive phenotype (Figure S6D,E), implying that EV‐transferred ACLY exerted its effects primarily by promoting fatty acid biosynthesis. To confirm this hypothesis, we supplemented cultured monocytes with acetate, an alternative precursor of lipogenic acetyl‐CoA through the action of acetyl‐CoA synthetase [40]. As predicted, acetate supplementation markedly rescued immunosuppressive differentiation of ACLY‐deficient monocytes, which was accompanied by enhanced lipid accumulation (Figure 4C,D).

To determine which specific metabolite(s) drove the observed immunosuppressive phenotype, we employed multiple pharmacological inhibitors targeting sequential enzymes within the fatty acid metabolic pathway. Following ACLY, acetyl‐CoA carboxylase (ACC) converts acetyl‐CoA to malonyl‐CoA, and fatty acid synthase (FASN) condenses these intermediates to generate palmitic acid, which forms palmitate in the basic cytosol. After that, stearoyl‐CoA desaturase (SCD) introduces a double bond to generate monounsaturated fatty acids (MUFAs), and elongation of very long‐chain fatty acid proteins (ELOVLs) can extend either saturated or SCD‐produced MUFAs into long‐chain species [43] (Figure 4E). By using inhibitors that selectively block these steps, we aimed to identify which metabolite(s) were critical for the immunosuppressive effects. Please note that THP1 and Raw264.7 cell lines were chosen in place of PBMC‐derived monocytes for the following biochemical analysis due to their stable growth, well‐characterized differentiation, and reproducible immune responses under controlled conditions [44]. Intriguingly, we found that ACLY‐induced immunosuppression was abolished by inhibitors of ACLY (SB204990), ACC (firsocostat), and FASN (C75), but not by inhibitors of SCD (A939572), or ELOVL (CAY10499), indicating that metabolite(s) synthesized up to the palmitate stage are essential for the immunosuppressive activity (Figure 4F,G and Figure S7A). Consistent with this notion, the compromised upregulation of immune checkpoint proteins caused by EVs with little ACLY could be rescued by supplementation with palmitate (PA) but not oleates (OA) (Figure 4H,I and Figure S7B). Moreover, the intracellular palmitate level was positively correlated with EV‐transferred ACLY (Figure 4J,K), and exogenous palmitate upregulated the expression of immune checkpoint proteins in a dose‐dependent manner (Figure 4L and Figure S7C). These findings collectively demonstrate that ACLY‐encapsulated EVs drive TAM polarization and immunosuppression by enhancing palmitate biosynthesis.

### 2.5 EV‐Transferred ACLY Promotes the Palmitoylation and Stability of CD276 and PD‐L1

**FIGURE 5:**
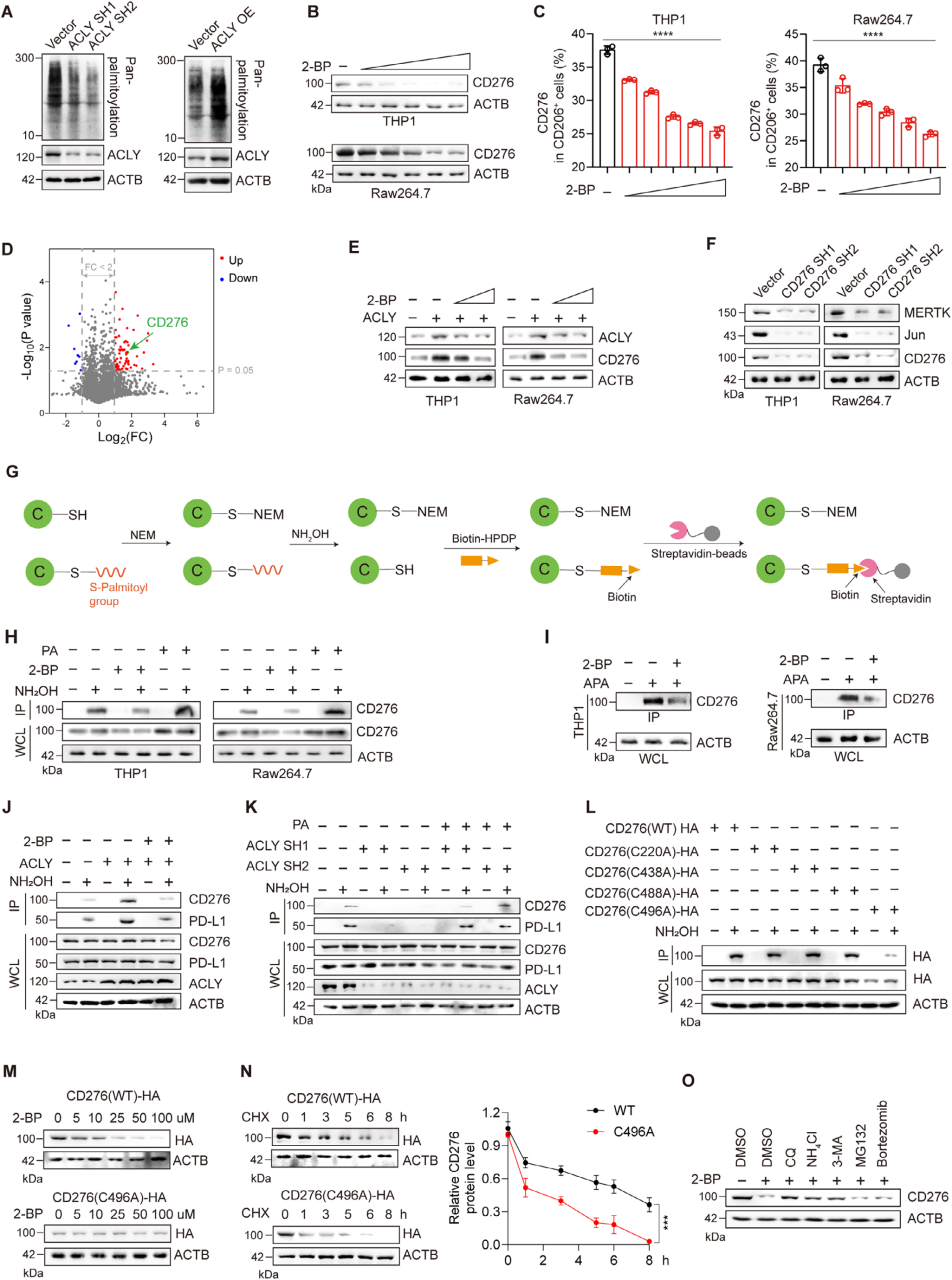
EV‐enriched ACLY drives palmitoylation of immune checkpoint proteins. (A) Pan‐palmitoylation levels in macrophages incubated with 5 µg EVs secreted from HepG2 cells with or without ACLY depletion or overexpression for 4 days. Representative of *n* = 3 independent experiments. (B,C) Immunoblot (B) and flow cytometry (C) analysis of CD276 expression in THP1 and Raw264.7 cells treated without or with increasing doses of 2‐BP (10–100 µm) for 24 h. One‐way ANOVA with Tukey's HSD test. (D) The volcano plot displays differentially palmitoylated proteins in monocytes following treatment with EVs derived from either ACLY‐overexpressing cells (experimental group) or normal cells (control group). Fold change (FC) was calculated as the ratio of the experimental group to the control group. (E) Immunoblot analysis of CD276 expression in THP1 and Raw264.7 cells incubated with 5 µg EVs derived from HepG2 cells without or with ACLY overexpression for 4 days, followed by treatment without or with 50–100 µm 2‐BP for 24 h. Representative of *n* = 3 independent experiments. (F) Levels of indicated proteins in THP1 and Raw264.7 cells transfected without or with two independent shRNAs depleting CD276. Representative of *n* = 3 independent experiments. (G) Schematic illustration of the ABE palmitoylation assay. Free cysteine thiols were blocked with N‐ethylmaleimide (NEM), then palmitoyl groups of examined proteins were removed by hydroxylamine (NH_2_OH), and the newly exposed cysteines were biotinylated by biotin‐HPDP, allowing enrichment of palmitoylated proteins using streptavidin beads. (H,I) Palmitoylation levels of CD276 in THP1 cells were detected by the ABE (H) and Click‐iT labeling assay (I). For the ABE assay, THP1 cells were treated without or with 100 µm 2‐BP or 500 µm PA, in the presence or absence of 0.5 m NH_2_OH. For the Click‐iT labeling assay, THP1 cells were treated without or with 100 µm 15‐azido‐pentadecanoic acid (APA) and/or 100 µm 2‐BP. Representative of *n* = 3 independent experiments. (J) CD276 and PD‐L1 palmitoylation in THP1 cells coculturing with 5 ug EVs derived from HepG2 cells with or without ACLY for 4 days, treated with 100 um 2‐BP for 24 h, followed with bafilomycin A1 (100 nm) and MG132 (10 um) for 2 h detected by ABE assay. Representative of *n* = 3 independent experiments. (K) CD276 and PD‐L1 palmitoylation in THP1 cells coculturing with 5 ug EVs derived from HepG2 cells with or without ACLY for 4 days, treated with 500 um PA for 24 h, followed with bafilomycin A1 (100 nm) and MG132 (10 um) for 2 h detected by ABE assay. Representative of *n* = 3 independent experiments. (L) Palmitoylation levels of CD276‐WT and indicated mutants in THP1 cells measured by the ABE assay. Representative of *n* = 3 independent experiments. (M) Exogenous expression of HA‐tagged CD276 wild‐type (WT) or C496A mutant in THP1 cells treated without or with increasing doses of 2‐BP for 24 h. Representative of *n* = 3 independent experiments. (N) The half‐lives of exogenous CD276 (WT) and CD276 (C496A)‐HA were determined by CHX (100 µm)‐chase assay for indicated periods of time. Relative levels of HA‐tagged proteins normalized to beta‐actin (ACTB) before CHX treatment were set as 1.0. Two‐tailed unpaired Student's *t*‐test. (O) Immunoblot analysis of CD276 expression in THP1 cells treated with dimethyl sulfoxide (DMSO) control or indicated inhibitors for 24 h. CQ, chloroquine. 3‐MA, 3‐methyladenine. Representative of *n* = 3 independent experiments. Data are presented as mean ± SD and represent three biological replicates. ***p < 0.001, ****p < 0.0001.

Given the unique role of palmitate in mediating immunosuppression, we speculated that palmitate‐induced protein palmitoylation of specific cysteine(s) might regulate the expression of immune checkpoint molecules. Based on this hypothesis, we examined whether EVs transferred ACLY could modulate protein palmitoylation levels in monocytes. Using a pan‐palmitoylation assay, we found that coculturing monocytes with EVs derived from ACLY‐overexpressing HCC cells significantly enhanced global protein palmitoylation (Figure 5A and Figure S8A). To test this palmitoylation of these immune checkpoint molecules, we analyzed the SwissPalm database (https://swisspalm.org/proteins) documenting protein palmitoylation events. This analysis revealed that PD‐L1 and CD276, but not MERTK, were likely palmitoylated proteins. Notably, PD‐L1 palmitoylation has been previously demonstrated to maintain its protein stability [45, 46, 47]. To determine whether CD276 is similarly palmitoylated and stabilized, we treated monocytic cell lines with 2‐bromopalmitate (2‐BP), a pan‐palmitoylation inhibitor. As expected, 2‐BP treatment led to a dose‐dependent reduction in CD276 expression (Figure 5B,C and Figure S8B). Furthermore, palmitoylomic analysis revealed that ACLY‐containing EVs promoted palmitoylation of CD276 (Figure 5D). Moreover, the upregulation of CD276 induced by EV‐transferred ACLY was reversed by 2‐BP treatment (Figure 5E and Figure S8C). Although MERTK itself is unlikely to be palmitoylated, CD276 transcriptionally upregulated MERTK via the transcription factor Jun (Figure 5F and Figure S8D) as previously reported [3]. Therefore, all three immune checkpoint proteins can be directly or indirectly regulated by palmitoylation events.

Next, we conducted the acyl‐biotin exchange (ABE) assay in which thiol groups of free cysteines are irreversibly blocked by N‐ethylmaleimide (NEM), whereas palmitoylated cysteines are cleaved by hydroxylamine (NH_2_OH) and subsequently biotinylated. Therefore, palmitoylated protein can be enriched using streptavidin beads (Figure 5G). Using this approach, we confirmed that CD276 was palmitoylated in both THP1 and Raw264.7 cells (Figure 5H and Figure S8E). In line with this finding, exogenous labeling with 15‐azido‐pentadecanoic acid (APA), a clickable probe for detecting protein palmitoylation, further verified that CD276 was palmitoylated (Figure 5I and Figure S8F). We then examined whether EV‐transferred ACLY regulated PD‐L1 and CD276 palmitoylation. Indeed, EV‐containing ACLY significantly enhanced PD‐L1 and CD276 palmitoylation that were efficiently suppressed by 2‐BP (Figure 5J and Figure S8G). In contrast, ACLY depletion in source HCC cells nearly abolished EV‐induced PD‐L1 and CD276 palmitoylation, which were otherwise restored by palmitate supplementation (Figure 5K and Figure S8H).

Whereas the palmitoylation site of PD‐L1 has been previously identified [47], the predicted palmitoylation site of CD276 has not yet been validated. To address this, we generated a panel of CD276 mutants in which the predicted palmitoylated cysteines were individually mutated to alanine. Compared with wild‐type (WT) CD276, the C496A mutant exhibited a marked reduction in CD276 palmitoylation (Figure 5L and Figure S8I), indicating that EV‐transferred ACLY promotes CD276 palmitoylation specifically at C496. This observation is consistent with the structural topology of CD276, as C496 resides within its short cytoplasmic tail [48]. We next investigated whether palmitoylation at this site contributes to CD276 protein stability. As expected, 2‐BP treatment accelerated the degradation of WT CD276 but not the C496A mutant (Figure 5M and Figure S8J). Moreover, the C496A mutant exhibited a faster reduction in protein abundance than WT CD276 in cycloheximide (CHX) chase assays (Figure 5N and Figure S8K). To determine the degradation pathway of depalmitoylated CD276, monocytic cells were respectively treated with the proteasomal inhibitor MG132 or bortezomib, the lysosomal inhibitor chloroquine (CQ), 3‐methyladenine (3‐MA), or ammonium chloride (NH_4_Cl). Whereas the latter three inhibitors rescued the 2‐BP‐induced CD276 degradation, MG132 or bortezomib had no significant effect on CD276 expression (Figure 5O and Figure S8L), indicating that CD276 palmitoylation prevents its degradation primarily through the lysosomal pathway. Collectively, these findings demonstrated that EV‐transferred ACLY promotes the palmitoylation and stabilization of multiple immune checkpoint proteins.

### 2.6 CD81‐Coated Liposomal Vesicles Mimic the Targeting Specificity of HCC‐Derived EVs

To demonstrate that EV‐transferred ACLY is sufficient to confer immunosuppression, we sought to generate a liposomal vesicular system capable of encapsulating ACLY as an internal cargo and mimicking the targeting specificity of HCC‐derived EVs. It was recently reported that LVs can be tailored to recapitulate the membrane composition and size of natural EVs based on DNA origami technology [49]. By incorporating DNA nanostructures as coprecipitates of crude LVs, tight size control and similar membrane composition can be achieved upon gradient purification of LV fractions with ∼150 nm diameter, which closely resembled natural EVs (Figure S9A). Next, uniformly‐dispersed LVs were characterized by TEM, nanoparticle tracking analysis, and zeta potential measurement (Figure S9B–D), which validated the quality of EV‐mimicking LVs. Additionally, LVs can carry bioactive proteins both on their surface and within their core, resembling the protein cargo‐loading capability of EVs (Figure S9E). To validate this, immunoelectron and immunoblot analysis were performed to confirm the attachment of enhanced green fluorescent protein (EGFP) to the LV surface, or effective encapsulation of EGFP within LVs (Figure S9F,G).

**FIGURE 6:**
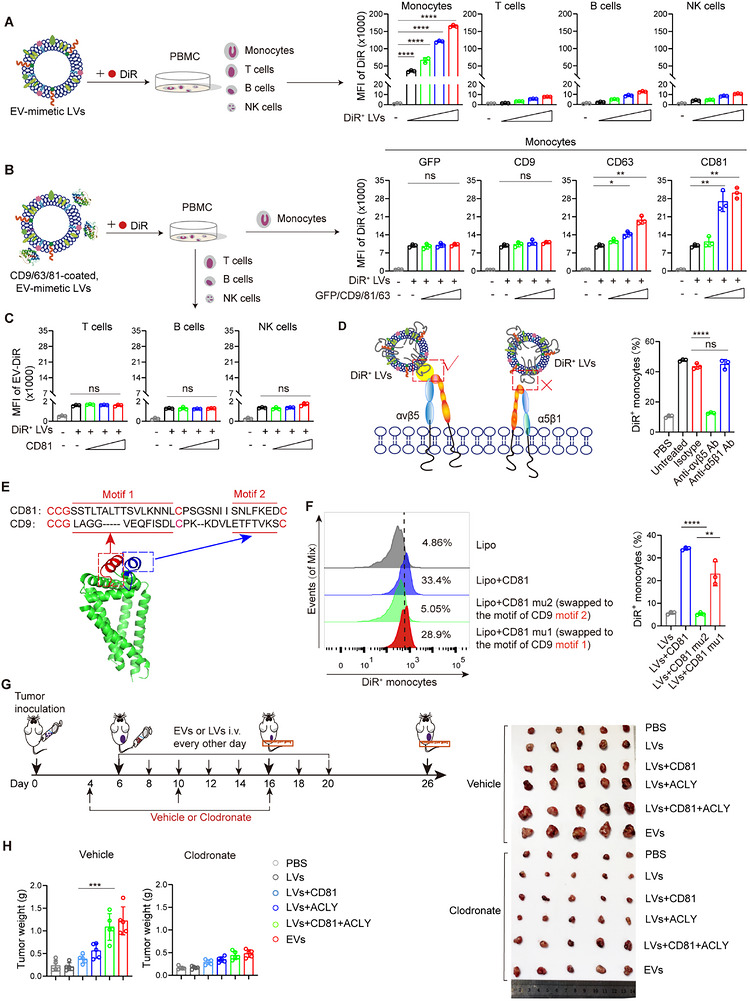
CD81‐coated LVs mimic the targeting specificity of EVs. (A) Delivery efficiency of various amounts (10–250 µg) of DiR‐labeled LVs to different immune cells in PBMCs after 1 h incubation. Left: schematic diagram. Right: Flow cytometry quantification of DiR‐positive fluorescence intensity in different immune cells incubated with EVs (*n* = 3). (B,C) Delivery efficiency of DiR‐labeled LVs coated with various amounts (10–50 µg) of different tetraspanin proteins (CD9/63/81) or GFP control to monocytes (B) or other immune cells (CD81 only, C) in PBMCs after 1 h incubation (*n* = 3). (D) The DiR positive fluorescence intensity in monocytes treated with different neutralizing antibodies (*n* = 3). (E) The sequence analysis of human CD81 and CD9. (F) The DiR positive fluorescence intensity in monocytes treated with indicated nanoparticles. Left: representative histograms. Right: representative histogram quantifications (*n* = 3). (G) Experimental design (left) and representative images (right) of Hepa1‐6‐derived subcutaneous tumors treated with PBS, EVs, or LVs carrying CD81 and/or ACLY via tail vein injection. Macrophage depletion was achieved using clodronate liposomes (*n* = 5). (H) Weight of harvested tumors from (G) (*n* = 5). Data are presented as mean ± SD. ns, not significant. *p < 0.05, **p < 0.01, ***p < 0.001, ****p < 0.0001 (one‐way ANOVA with Tukey's HSD test).

We then evaluated the uptake efficiencies of LVs by various immune cells. Briefly, LVs were supplemented to human PBMCs, or intravenously injected into mice followed by immune cell purification from hepatic tissues. Consistent with endogenous EVs, LVs preferentially fused with monocytes, whereas minimal fusion was observed with T cells, B cells, or NK cells (Figure 6A and Figure S10A,B). However, the extent to which endogenous EVs fused with monocytes clearly surpassed LVs, indicating that additional EV effector(s) were involved in targeting recipient cells. Tetraspanins were EV surface proteins frequently participated in EV biogenesis, cargo transport, and targeting selectivity to recipient cells [50, 51]. Among various tetraspanins, CD81, CD63, and CD9 are ubiquitously expressed on the EV surface [50]. Therefore, we generated LVs decorated respectively with the extracellular domain of CD81, CD63, and CD9 (referred to as CD81/CD63/CD9‐coated LVs), and tested their targeting specificities compared with control LVs decorated with EGFP. Intriguingly, CD81‐coated LVs showed highest, and CD63‐coated LVs showed slightly enhanced, uptake efficiencies toward PBMC‐derived monocytes but not other immune cell populations (Figure 6B,C). These findings were further corroborated by in vivo data that CD81‐coated LVs preferentially fused with monocytes in the mouse liver, followed by spleen and blood with less efficiency, whereas other immune cell populations remained largely unaffected (Figure S10C–E).

To explore why CD81 contributed to the EV targeting and uptake process, we investigated the potential role of integrin receptors expressed on the surface of monocytes and macrophages. Integrins are heterodimeric receptors that mediate cell–cell adhesion and are known to interact with tetraspanins [52, 53, 54, 55]. Among the integrin receptor family, αvβ5 is highly expressed on monocyte‐derived macrophages and plays a critical role in phagocytosis [55, 56]. Previous studies have shown that CD81 but not CD9 can recognize integrin αvβ5 [55], promoting the selective binding of EVs to macrophages. To validate the potential role of integrin αvβ5 in EV uptake, we treated PBMC‐derived monocytes with αvβ5‐neutralizing antibodies. Such treatment significantly reduced the uptake efficiency of CD81‐LVs, whereas neutralizing another commonly‐expressed integrin α5β1 exhibited little effects (Figure 6D). In addition, we performed structural analysis of CD81 [52] by creating mutations respectively in two motifs of its extracellular domain that dictate the sequence difference between CD81 and CD9 (Figure 6E). We found that switching the motif 2 of CD81 to the corresponding region of CD9 markedly reduced the uptake efficiency of CD81‐LVs, whereas switching the motif 1 of CD81 failed to do so (Figure 6F). These data suggest that CD81 interacts with integrin αvβ5 through its extracellular motif 2, thereby promoting the recognition and internalization of EVs by monocytes and macrophages.

### 2.7 ACLY‐Loaded, CD81‐Coated LVs Promote Immunosuppression and Tumor Growth

Given the similar targeting specificities of CD81‐coated LVs and endogenous EVs, we utilized this reconstitution system to validate the tumor‐promoting role of ACLY as a critical EV cargo during HCC progression. First, C57BL/6J mice were inoculated with Hepa1‐6 cells, followed by intravenous injection of various LVs or equal amounts of HCC‐derived EVs at indicated time points (Figure 6G, left panel). As a result, transferring ACLY within LVs (LVs+ACLY) partially enhanced tumor growth compared with the LV control group (Figure 6G, right panel, line 4 vs. line 2; Figure 6H and Figure S10F, left panels, sample 4 vs. sample 2). In contrast, CD81‐coated empty LVs (LVs+CD81) failed to alter the tumor growth profile (Figure 6G, right panel, line 3 vs. line 2; Figure 6H and Figure S10F, left panels, sample 3 vs. sample 2). Importantly, ACLY‐loaded, CD81‐coated LVs (LVs+CD81+ACLY) maximized tumor growth resembling the effects of HCC‐derived EVs (Figure 6G, right panel, line 5 vs. line 6; Figure 6H and Figure S10F, left panels, sample 5 vs. sample 6). These results demonstrated that ACLY is the functional cargo of HCC‐derived EVs that sufficiently drives tumor progression.

Next, immune profiling of harvested tumor tissues revealed that administration of ACLY‐encapsulated, CD81‐coated LVs markedly upregulated the expression of immune checkpoint proteins MERTK, CD276, and PD‐L1 in CD206^+^ TAMs (Figure S10G), recapitulating the immunosuppressive polarization pattern observed in tumors treated with HCC‐derived EVs. To prove that TAMs were directly responsible for EV‐ or LV‐ accelerated tumor growth, we depleted macrophages by the injection of clodronate liposomes, which strongly undermined EV‐ or LV‐ promoted HCC tumor growth (Figure 6G,H and Figure S10F, right panels).

Importantly, the ketogenic diet shifts metabolism toward fatty acid oxidation and ketogenesis, leading to reduce the expression of lipogenic enzymes [57], including ACLY (Figure S11A–D). ACLY‐encapsulated LVs markedly upregulated the expression of immune checkpoint proteins MERTK, CD276, and PD‐L1 in CD206+ TAMs (Figure S11B,C). The depletion of macrophages inhibited tumor progression accompanied by reduced TAM abundance (Figure S11B,C). These findings underscored the critical role of TAMs in promoting HCC progression, and confirmed that EV‐transferred ACLY facilitated tumor growth primarily through inducing immunosuppressive TAMs.

### 2.8 Protein Levels of ACLY in Plasma EVs Correlate With HCC Progression

**FIGURE 7:**
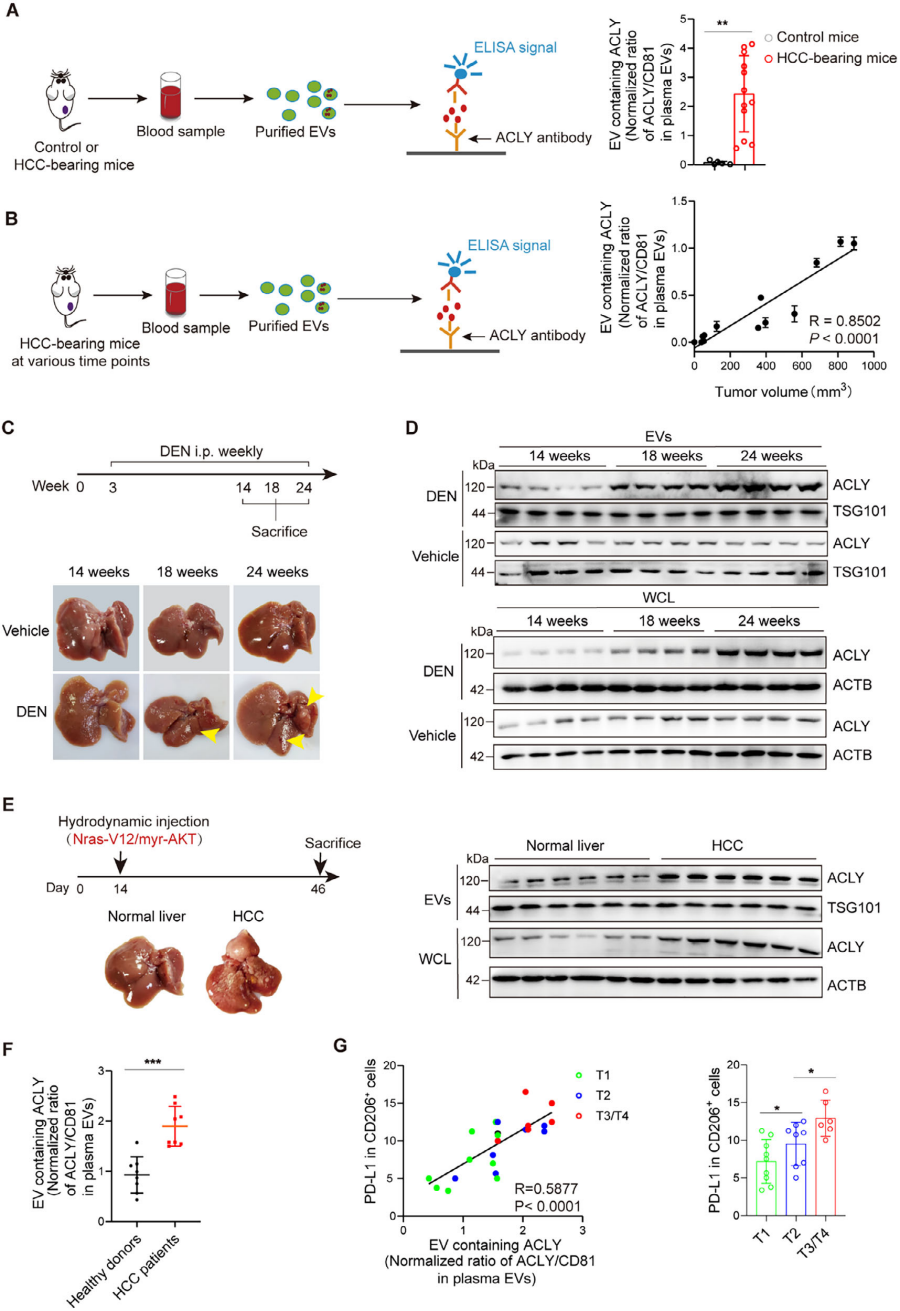
ACLY levels of plasma EVs correlate with HCC tumor progression. (A) Experimental design (left) and ELISA quantification (right) of ACLY levels in plasma EVs collected from SCID mice with or without HepG2‐derived HCC tumors (*n* = 12). (B) Experimental design (left) and correlations between ACLY levels in plasma EVs collected from C57BL/6J mice with Hepa1‐6‐derived HCC tumors at various time points and corresponding tumor volumes (right) (*n* = 3 per time point). (C,D) Schematic diagram of experimental design (C, top panel), representative liver images (C, bottom panel), and immunoblot analysis of hepatic whole cell lysate (WCL) and plasma EVs (D) collected from the HCC model created by intraperitoneally (i.p.)‐injected 50 mg/kg diethylnitrosamine (DEN). (E) Schematic diagram of experimental design and representative liver images (left), and immunoblot analysis of liver tissues and plasma EVs (right) collected from the HCC model created by hydrodynamically injected Nras‐V12 and myristoylated AKT (myr‐AKT). Normal liver tissues and plasma EVs harvested from noninjected mice were used as negative control. (F) ELISA quantification of ACLY levels of plasma EVs collected from healthy donors and HCC patients coenrolled during the same time period (*n* = 8 for each group). (G) Correlations between ACLY levels of plasma EVs and PD‐L1^+^CD206^+^ TAMs in T1 (*n* = 9), T2 (*n* = 8), T3/4 (*n* = 6) grade HCC tissues from a local patient cohort (*n* = 23). Data are presented as mean ± SD. Unpaired two‐tailed Student's *t*‐test (A,F) and one‐way ANOVA with Tukey's HSD test (G). ns, not significant. *p < 0.05, **p < 0.01, ***p < 0.001.

Based on the observation that EV‐containing ACLY promotes HCC tumor growth, we sought to determine whether HCC‐released EVs contain more ACLY proteins when tumors develop to higher grades, thereby serving as a potential diagnostic marker for HCC progression. Moreover, previous studies have shown that the protein content of tumor‐released EVs is proportional to the overall tumor burden [58, 59], which may facilitate the detection of ACLY in HCC‐derived EVs purified from plasma despite the presence of background EVs derived from other blood–borne cells. To test this hypothesis, we first collected plasma samples from HCC‐bearing mice for EV isolation, and subsequently analyzed the EV levels of ACLY (hereinafter, normalized to CD81 levels to correct for differences in EV quantities) using the enzyme‐linked immunosorbent assay (ELISA). We found that ACLY was readily detectable in circulating EVs harvested from HCC‐bearing mice, but almost undetectable in circulating EVs from control mice (Figure 7A). Moreover, ACLY levels of plasma EVs closely correlated with sizes of HCC tumors harvested at different time points, indicating that larger tumors likely secreted EVs carrying more ACLY proteins as their cargo (Figure 7B).

Next, we went on to examine the relationship between ACLY levels of HCC‐derived EVs and tumor progression, using a diethylnitrosamine (DEN)‐induced autochthonous HCC mouse model that recapitulates the full course of tumor initiation and progression. HCC tumor and plasma samples were collected at indicated time points after DEN treatments (Figure 7C), and plasma EV‐containing ACLY was quantified relative to the EV marker protein TSG101. As expected, we observed a parallel increase in ACLY levels of plasma EVs and intratumoral ACLY levels as HCC progressed (Figure 7D). This phenotype was reproduced in a more rapid autochthonous HCC mouse model induced by hydrodynamic injections of mutant NRAS and AKT (Figure 7E). Therefore, ACLY levels were significantly enhanced in EVs derived from high‐grade HCC tumors with elevated ACLY levels, regardless of carcinogenic origin. To further verify this correlation in clinics, we collected paired plasma and tumor samples from a local HCC patient cohort. Indeed, plasma EVs from HCC patients exhibited higher ACLY levels compared with plasma EVs from healthy donors (Figure 7F). Moreover, the amounts of tumor‐infiltrating PD‐L1^+^CD206^+^ TAMs positively correlated with levels of plasma EV‐containing ACLY, particularly in patients with high‐grade HCC (Figure 7G). These data collectively suggested that ACLY levels of circulating EVs can potentially be used as a diagnostic indicator for HCC progression.

### 2.9 CD81‐Coated LVs Loaded With ACLY Inhibitors Restrain HCC Progression and Improve Immunotherapeutic Efficacy

Given the critical role of EV‐containing ACLY in promoting immunosuppressive TAMs and tumor growth, we attempted to develop a relevant therapeutic strategy to treat HCC. SB204990 is a specific ACLY inhibitor showing promise in the preclinical setting [39]. However, systemic administration of SB204990 led to multiple side effects including severe weight loss, which limited its clinical translation [60, 61]. To overcome this, we loaded SB204990 into CD81‐coated LVs, generating SB204990‐encapsulated, CD81‐coated LVs (SCLVs) to confer the TAM‐targeting specificity to this inhibitor and reduce its side effects. As expected, repeated SCLVs treatments caused no significant weight loss in mice, demonstrating that this delivery system mitigated the side effects associated with systemic ACLY inhibition (Figure S12A).

**FIGURE 8:**
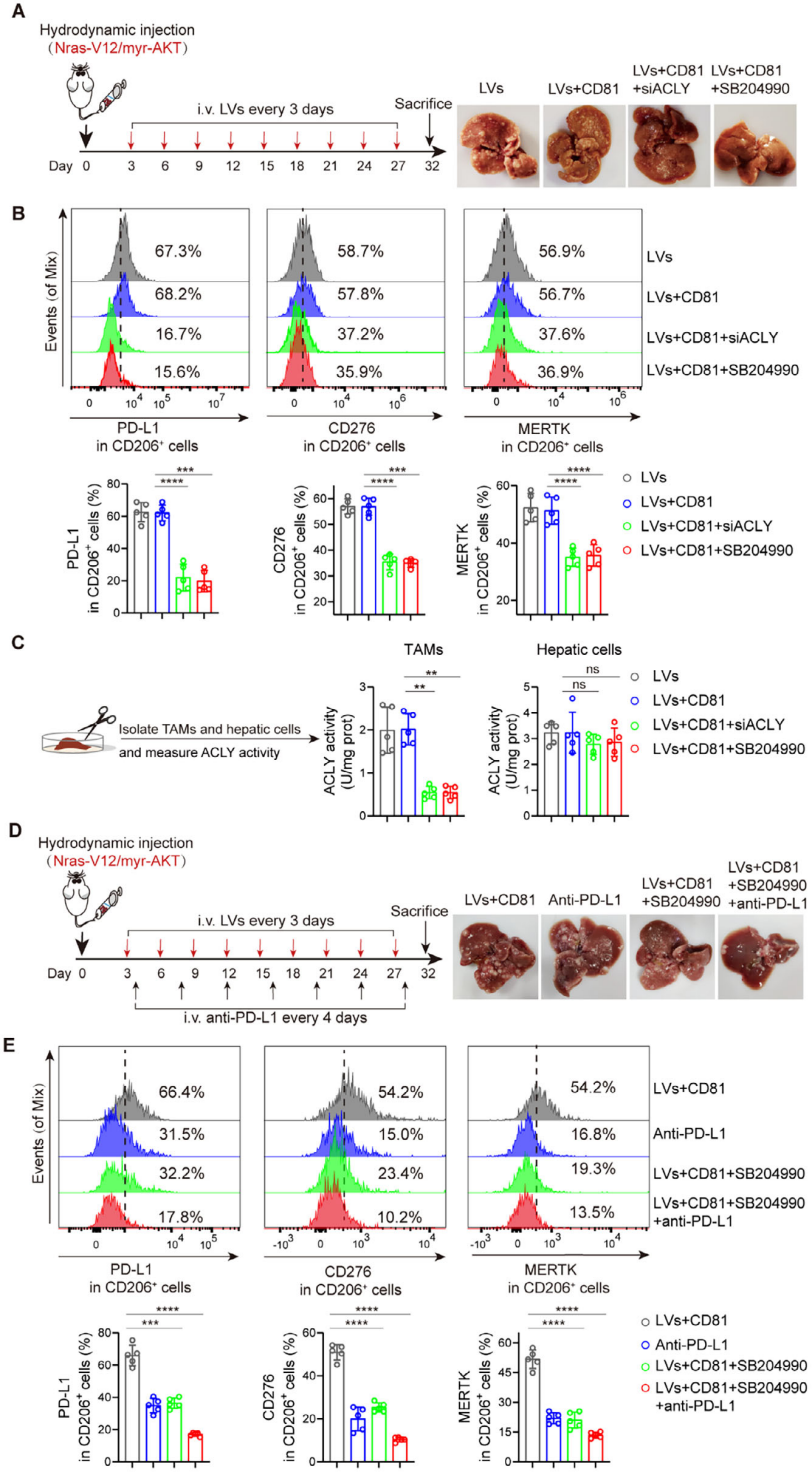
CD81‐coated LVs loaded with ACLY inhibitors enhance immunotherapy efficacy and restrain HCC progression. (A) Schematic diagram (left) and representative livers (right) harvested from the HCC model created by hydrodynamically injected Nras‐V12 and myr‐AKT, i.v. treated with LVs loaded without or with CD81, the ACLY inhibitor SB204990, or small interfering RNAs depleting ACLY (siACLY). (B) Representative histogram (upper panel) and quantification (lower panel) of flow cytometry analysis of immunosuppressive TAMs isolated from mouse livers indicated in (A) (*n* = 5). (C) Measurement of the ACLY enzymatic activity in TAMs and hepatic cells isolated from mouse livers as indicated in (A) (*n* = 5). (D) Schematic diagram (left) and representative livers (right) harvested from the HCC model created by hydrodynamically injected Nras‐V12 and myr‐AKT, i.v. treated without or with anti‐PD‐L1 antibodies and/or CD81‐coated LVs loaded without or with SB204990. (E) Representative histogram (upper panel) and quantification (lower panel) of flow cytometry analysis of immunosuppressive TAMs isolated from mouse livers indicated in (D) (*n* = 5). Data are presented as mean ± SD. ns, not significant. ***p < 0.001, ****p < 0.0001 (one‐way ANOVA with Tukey's HSD test).

Moreover, in the autochthonous HCC model induced by mutant NRAS and AKT, treatments with SCLVs or LVs encapsulating small‐interfering RNAs depleting murine ACLY (siACLY) inhibited tumor progression accompanied by reduced TAM abundance (Figure 8A,B and Figure S12B). Further analysis revealed that SCLVs inhibited the ACLY enzymatic activity and accumulation of intracellular lipid droplets specifically in TAMs, but not in pan‐hepatic cells mainly composed of hepatocytes (Figure 8C and Figure S12C,D). Importantly, we then assessed the potential effects of combination therapy using SCLVs and anti‐PD‐L1 antibodies in autochthonous HCC‐bearing mice, given the limited clinical efficacy of anti‐PD‐1/PD‐L1 therapies against HCC [1, 62], As expected, this combination therapy exhibited clear synergistic effects, with a stronger inhibition of tumor growth and a greater attenuation in immunosuppressive TAMs compared to either monotherapy alone (Figure 8D,E and Figure S12E). These findings demonstrated that targeting ACLY‐high TAMs in combination with anti‐PD‐1/PD‐L1 antibodies enhance the efficacy of immune checkpoint blockade and achieve a better therapeutic response in HCC.

## 3 Discussion

The differentiation of monocytes into TAMs plays a crucial role in shaping the immune landscape of TME. Monocytes can differentiate into DCs or macrophages [63], with each cell type playing a distinct role in tumor immunity [64]. DCs are essential for initiating the adaptive anti‐tumor immune responses by presenting antigens to cytotoxic T lymphocytes [63, 65]. On the contrary, monocyte‐derived macrophages can be polarized into anti‐ or protumorigenic subsets, depending on particular signals received from the TME [66]. The balance between anti‐ and protumorigenic macrophages is a key determinant of cancer progression and effectiveness of immunotherapies [65, 67, 68]. In the context of HCC tumors, monocyte differentiation is heavily regulated by the surrounding microenvironment [67, 68]. Tumor cells release a complex array of EVs, metabolic signals, and cytokines that dictate the fate of monocyte‐derived macrophages [66, 69]. For instance, CD40 signaling drives pro‐inflammatory macrophage polarization by coordinating reduced β‐nicotinamide adenine dinucleotide (NADH) and fatty acid metabolism [70]. Moreover, EVs carrying pyruvate kinase M2 or Yes‐associated protein create an immunosuppressive niche by promoting the infiltration of protumorigenic macrophages [25, 59].

Emerging evidences indicate that deregulated lipid metabolism, partially reprogrammed by EV‐encapsulated lipids and metabolic enzymes, serve as key regulators of immune responses in multiple diseases [18, 58, 70]. For example, exosomal palmitate induces abnormal fatty acid metabolism in Kupffer cells and exacerbates fatty liver disease [18]. Similarly, sphingolipids including ceramides and sphingosine‐1‐phosphate regulate the metabolism, distribution, and activation of tumor‐infiltrating T lymphocytes [71]. However, current knowledge about how metabolic signals in the TME influence macrophage fate decision remains limited. Our study highlights the role of tumor‐derived EVs, specifically those enriched with the lipogenic enzyme ACLY, in driving the immunosuppressive polarization of TAMs in HCC. EV‐containing ACLY reprograms macrophage metabolism by promoting palmitate biosynthesis, which is essential for the protumorigenic function of TAMs (Figure S13). Enhanced palmitate levels promote the palmitoylation and stabilization of immune checkpoint proteins PD‐L1 and CD276, in line with previous reports that palmitoylation can shield proteins from ubiquitination and proteasomal degradation, or protect them from autophagy‐mediated lysosomal degradation [72].

To further validate our results, we developed a liposomal system using CD81‐coated LVs to selectively fuse with TAMs, resembling the targeting specificity of endogenously produced EVs. It has been reported that multiple factors regulate the EV targeting specificity, including the lipid composition, glycosylation patterns, and membrane receptors of EVs [50, 73]. For example, the Notch receptor‐ligand interaction promotes EV‐mediated neuron‐to‐neuron communications [74, 75]. N‐linked glycan on the EV surface improves the receptor‐mediated targeting of DCs [76]. Tetraspanins regulate EV biogenesis, cargo sorting, and EV uptake by various target cells [50]. Consistent with these findings, our data revealed that the tetraspanin protein CD81 governs the targeted fusion between HCC‐derived EVs and recipient monocytes/macrophages. Therefore, we anchored the extracellular domain of CD81 onto the LV surface, generating biomimetic LVs with EV‐comparable targeting specificities. By encapsulating the ACLY inhibitor SB204990 in CD81‐coated LVs, we efficiently targeted the immunosuppressive function of TAMs without affecting other cell populations. Therefore, this approach safely improved the efficacy of anti‐PD‐L1 immunotherapy in HCC.

Overall, our study offers compelling evidence that targeting EV‐transferred ACLY in TAMs represents a safe and promising therapeutic strategy for treating HCC. HCC‐derived EVs are specifically transmitted to monocytes and macrophages in vivo, driving their palmitate biosynthesis and protumorigenic differentiation. These findings provide new insights into the metabolic demand for TAM differentiation and underscore the therapeutic potential of targeting ACLY in HCC‐associated TAMs for enhanced anti‐PD‐1/PD‐L1 efficacy. Our results indicate that higher levels of exosomal ACLY are associated with increased HCC risk. This finding has clinical relevance because plasma derived exosomes are already used in clinical diagnostics, and previous studies have shown that the liver is a major source of plasma EVs [77]. However, isolating liver‐specific EVs directly remains difficult. The liver's high fat and fibrotic tissue make extraction challenging, and the need for fresh samples adds to this difficulty [78]. Future studies should focus on improving methods for EV isolation and exploring their role in tumor progression.

Moreover, the metabolomic analysis revealed EV‐transferred ACLY elevates the levels of palmitic acid in macrophage. These studies indicated the significance of palmitate in mediating exosome‐driven ACLY transfer and immunosuppression. This defines a cell‐intrinsic metabolic mechanism. While the role of receptors like CD36 in the uptake of extracellular palmitate is a distinct scientific question, our work conclusively shows that the intracellular de novo synthesis pathway mediated by transferred ACLY is a potent and self‐sufficient driver of immune evasion. Future studies are warranted to decipher extensive roles of metabolic deregulation in tumor immunity, and its broader implications for improving cancer treatment outcomes.

## 4 Method

### 4.1 Cell Culture

HHL‐5, SNU449, and THP1 cells were cultured in RPMI‐1640 (Corning, 10‐040‐CV), supplemented with 10% fetal bovine serum (FBS, TransGen Biotech, FS301‐02) and 1% penicillin/streptomycin (Thermo Fisher Scientific, 15070‐063). HEK‐293T, Hepa1‐6, and Raw264.7 cells were cultured in Dulbecco's modified Eagle Medium (DMEM) (Corning, 10‐013‐CV) with 10% FBS and 1% penicillin/streptomycin. HepG2 cells were cultured in DMEM medium with nonessential amino acid (NEAA) (Procell, PM150410), supplemented with 10% FBS and 1% penicillin/streptomycin. Cell lines were verified with STR authentication and tested twice per month to avoid mycoplasma contamination, and primary cell cultures were confirmed mycoplasma‐free 2 h before experiments. All cells were employed within a passage range of 3 to 12 to ensure genetic stability and phenotypic consistency as indicated in Table S1.

THP1 cells were treated with 100 ng/mL phorbol 12‐myristate 13‐acetate (PMA, Selleck, S7791) for 24 h, followed by 24 h recovery for macrophage differentiation, and then stimulated with 20 ng/mL IL‐4 (Peprotech, 200‐04) to induce M2‐like polarization if needed. Raw264.7 cells were cultured with 20 ng/mL IL‐4. Cells were treated with the following reagents: SB204990 (MCE, HY‐16450), C75 (MCE, HY‐12364), Firsocostat (Aladdin, F421601), A939572 (Macklin, A794424), CAY10499 (APExBIO, CAY10499), 2‐BP (MCE, HY‐111770), palmitic acid (MCE, HY‐N0830), oleic acid (Aladdin, O431503), cycloheximide (MCE, HY‐12320), 15‐azido‐pentadecanoic acid (MCE, HY‐151656).

### 4.2 Isolation of Immune Cell Populations From Human Peripheral Blood

Peripheral blood from healthy human donors was obtained from the Guangzhou Blood Center. The study protocol was approved by the Institute Research Medical Ethics Committee at Sun Yat‐Sen University. PBMCs were isolated using standard density gradient method. Next, human monocytes were isolated from PBMCs by magnetic cell sorting method using anti‐CD14 microbeads (Miltenyi Biotec, 130‐050‐201). Monocyte‐derived macrophage differentiation in vitro was initiated by treatments with macrophage colony‐stimulating factor (M‐CSF, 50 ng/mL, Peprotech, 300‐25) for 6 days. Cells were subjected to flow cytometry analyses of CD206, MERTK, PD‐L1, and CD276. All applied antibodies were listed in Table S2.

### 4.3 Isolation of Murine Monocytes

For isolation of murine monocytes, bone marrow was extracted from the mouse bone cavity using a 1 mL syringe. After filtration and centrifugation at 1800 rpm for 10 min, red blood cells were lysed, and the remaining cells were resuspended in complete medium supplemented with murine M‐CSF (10 ng/mL, Peprotech, 315‐02) for 5–6 days. Cells were subjected to flow cytometry analyses of CD206, MERTK, PD‐L1, and CD276. All applied antibodies were listed in Table S2.

### 4.4 Isolation of Immune Cell Populations From Mouse Liver or Tumor Tissue

For isolation of different immune cell populations from murine liver or tumor, tissue samples were cut into pieces and digested with RPMI 1640 containing 0.75 mg/mL type IV collagenase (Sigma, C5138) and DNase I (Sigma, DN25) for 45 min. The solution was then filtered through a 70 µm strainer, and cells were subjected to density gradient centrifugation using Ficoll. The isolated cells were then stained and analyzed by flow cytometry, and gating strategy was provided in Figure S14. Note that CD206^+^ cells were manually sorted using anti‐biotin microbeads (Miltenyi Biotec, 130‐090‐485) coupled with biotinylated anti‐CD206 antibodies. All applied antibodies were listed in Table S2.

### 4.5 Isolation of EVs

EVs were isolated and purified according to the MISEV guidelines in 2018 [34, 35, 36]. Briefly, HCC cells (HepG2, SNU449, or Hepa1‐6) were grown to 80% confluence, washed 3 times with PBS, and cultured for 2 days in RPMI 1640 with 10% EV‐depleted serum. The supernatant was collected and centrifuged at 300 g for 10 min, followed by 2000 g for 10 min, and 12 000 g for 20 min to deplete cell debris. The supernatant was then filtered using a 0.22 µm filter membrane. After that, EVs were purified by ultracentrifugation at 100 000 g for 2 h and washed twice with PBS, followed by resuspension in PBS. The protein concentration of EVs was measured using the bicinchoninic acid (BCA) protein assay kit (Beyotime, P0011).

### 4.6 Synthesis of LVs

The biomimetic LVs were composed of dioleoylphosphatidylcholine (DOPC), dioleoylphosphatidylethanolamine (DOPE), dioleoylphosphatidylserine (DOPS), sphingomyelin, and cholesterol at a molar ratio of 21:17.5:14:17.5:30, which were dried overnight to form a homogeneous lipid mix. PBS buffer was then added and stirred in a water bath at 42°C for 30 min to fully hydrate the mix. The hydrated emulsion was sonicated to obtain a uniformly dispersed liposomal suspension. To differentiate heterogeneous sizes of LVs, brick‐like DNA structures were designed by applying DNA nanotechnology as previously described [79], in order to capture crude LVs with various sizes. Cholesterol‐modified DNA bricks were designed by assembling a three‐point star DNA structure with cholesterol at the end of each strand as the membrane anchor. These bricks were incubated with LVs at a molar ratio of 1:375 to attach to the liposomal surface. LVs ranging in diameter from 30 to 200 nm were separated by gradient centrifugation, and fractions with ∼150 nm diameter were eventually purified as EV‐mimicking LVs. After purification, attached DNA nanostructures were removed from LVs by DNase digestion.

### 4.7 Characterization of EVs and LVs

Endogenous EVs and biomimetic LVs were characterized by TEM, NTA, and zeta potential analysis as previously described [69]. Colloidal gold‐labeling immunoelectron microscopy and Western blot analysis were used to confirm that His‐tagged proteins could be loaded onto the LV surface via the DOGS‐NTA‐Ni molecule, as described in previous studies [79].

### 4.8 Cell Uptake Assay

For fluorescent labeling experiments, 100 µL EVs, LVs, or an equivalent volume of PBS control were respectively mixed with 5 mg/mL DiR (Thermo Fisher Scientific, D12731) and incubated at 37°C for 1 h. The stained vesicular mixtures were washed twice with PBS, followed by ultracentrifugation at 100 000 g for 2 h to remove unincorporated dyes. The pellets were washed and resuspended with PBS.

For coculture experiments in vitro, DiR‐labeled EVs or LVs were incubated with PBMCs at 37°C for 4 h, followed by staining with fluorescent antibodies specific to different immune cells including NK cells, T cells, B cells, monocytes, and macrophages before flow cytometry analysis. To identify the immune cells that took up EVs or LVs in vivo, DiR‐labeled EVs or LVs were injected into mice via the tail vein. At different time points (0.5, 1, 3 h, etc.), immune cell populations from blood, liver, spleen, or tumors were isolated, stained with fluorescent antibodies against various immune cell markers, and analyzed for DiR‐positive fluorescence intensity.

### 4.9 Coculture Assay

For direct coculture experiments, HCC cells or hepatocytes transfected with control shRNA, or shRNAs against ACLY, CAD, or TKT (see sequences in Table S3), as well as cells overexpressing ACLY were incubated with M‐CSF‐treated monocytes or macrophages. Cells were then harvested and subjected to flow cytometry analysis. For coculture of monocytes/macrophages with EV‐free supernatant, the culture supernatant from HCC cells or hepatocytes was subjected to ultracentrifugation to remove EVs, and the resultant EV‐free supernatant was used for incubation. For EV coculture assays, 5 µg EVs were isolated from the culture medium of HCC cells or hepatocytes by ultracentrifugation, and then incubated with 0.1 million monocytes/macrophages.

### 4.10 Electroporation

Total RNA and miRNA were isolated using Ambion mirVana miRNA Isolation Kit (Thermo Fisher Scientific, AM1561), and DNA were extracted with the DNA Extraction kit (Qiagen, 69554). 2 × 10^6^ monocytes were resuspended in 100 µL of freshly prepared electroporation buffer. Then, 30 µg nucleic acid (miRNA, DNA, or RNA) was added to the cell suspension and mixed gently to avoid bubble formation. Electroporation was performed using the Lonza 4D‐Nucleofector system (4D‐Nucleofector Core Unit: AAF‑1002B; 4D‑Nucleofector X Unit: AAF‑1002X). Following electroporation, the cells were incubated at 37°C for 10 min. Thereafter, 500 µL of prewarmed complete medium was added, and the cells were gently transferred into a 6‐well plate containing an additional 1.5 mL of prewarmed complete medium.

### 4.11 ABE Assay

THP1 or Raw264.7 cells were lysed in ice‐cold lysis buffer (50 mm Tris pH = 7.2, 150 mm NaCl, 1% NP‐40, 1 mm EDTA) supplemented with 20 mm NEM (Aladdin, E100553) and 1× protease inhibitor cocktail for 2 h at 4°C to completely block free thiols. The lysate was centrifuged at 16 000 × *g* for 15 min, and the protein concentration was determined. 1 mg protein was precipitated with methanol (800 µL)‐chloroform (200 µL)‐water (600 µL) (CM precipitations) to remove residual NEM. The pellet was dissolved in 100 µL 1% sodium dodecyl sulfate (SDS), followed by addition of 400 µL lysis buffer with PBS or 0.5 m hydroxylamine (NH_2_OH, Macklin, H790423) to promote depalmitoylation. After 1 h incubation at room temperature, proteins were reprecipitated with CM precipitations to remove NH_2_OH. The precipitate was redissolved in 100 µL 1% SDS, mixed with 400 µL lysis buffer containing 4 mm EZ‐Link biotin‐HPDP (N‐[6‐(biotinamido)hexyl]‐3′‐(2′‐pyridyldithio)propionamide, ThermoFisher, A35390), and incubated at room temperature for 1 h. Proteins were precipitated again with CM precipitations. The final pellet was dissolved in 100 µL lysis buffer with 1% SDS at 37°C for 15 min, diluted 1:10 with PBS, and incubated with 30 µL streptavidin beads overnight at 4°C. Beads were washed 4 times with lysis buffer, and bound proteins were eluted with 50 µL 2× SDS loading buffer containing β‐mercaptoethanol or dithiothreitol (DTT).

### 4.12 Click‐iT Labeling Assay

THP1 or Raw264.7 cells were treated with 100 µm APA (MCE, HY‐151656) for 8 h, and lysed in lysis buffer (50 mm Tris pH = 7.2–8, 150 mm NaCl, 1% NP‐40, and 1 mm EDTA) containing protease inhibitor cocktail for 2 h on ice. After centrifugation at 16 000 × *g* for 15 min, 200 µg protein was reacted with 100 µm dibenzocyclooctyne (DBCO)‐PEG4‐biotin (MCE, HY‐130809) for 1 h at room temperature. The reaction was stopped by CM precipitations. The pellet was dissolved in 100 µL of 1% SDS, isolated using streptavidin beads (Thermo Fisher Scientific, 20347), and then subjected to immunoblotting to detect palmitoylated proteins.

### 4.13 Detection of EV‐Containing ACLY in Plasma

For EV isolation from plasma, frozen plasma aliquots were thawed on ice. 200 µL plasma was diluted with PBS and subjected to sequential centrifugation: first at 2000 × *g* for 20 min to remove debris, then at 10 000 × *g* for 45 min at 4°C. The resulting supernatant was centrifuged at 100 000 × *g* for 2 h at 4°C to obtain EVs. The pellet was washed with PBS, recovered by another ultracentrifugation at 100 000 × g for 70 min at 4°C, and finally resuspended in 100 µL radioimmunoprecipitation assay buffer (RIPA) buffer with protease inhibitor cocktail (MCE, HY‐K0010). To detect EV‐containing ACLY or CD81 from human or mouse plasma, EVs were lysed in RIPA buffer, and ELISA was performed using human or mouse ACLY or CD81 ELISA kits (Raybiotech, ELH‐ACLY‐1; Taiclone, tcfe4100; LSBio, LS‐F55938; Cusabio, CSB‐EL004960MO).

### 4.14 Quantification of Free Fatty Acid (FFA) and Palmitic Acid

EVs were isolated from the culture medium of HCC cells by ultracentrifugation. Following coculture with macrophages, EV‐incubated macrophages were collected. FFA and palmitic acid levels were quantified using commercial colorimetric assay kits (FFA, Wako, 294‐63601; palmitic acid, COBIOBIO, CB21418). Sample protein concentrations were determined in parallel with a detergent‐compatible BCA protein assay. Final FFA and palmitic acid concentrations were normalized to the total protein content for comparative analysis.

### 4.15 Lipidomic Analysis

Lipids were extracted from purified macrophages using a chloroform:methanol:H_2_O (5:5:2) solution. After incubation at −20°C and centrifugation, the chloroform phase was collected, dried, and reconstituted for derivatization by conversion of fatty acids to fatty acid methyl esters. The derivatized lipids were analyzed by ultraperformance liquid chromatography tandem mass spectrometry (UPLC‐MS/MS). Data processing including peak detection and lipid identification was performed using the MS‐DIAL software.

### 4.16 Immunoblot

Harvested cells were lysed in RIPA buffer with protease inhibitor cocktail (MCE, HY‐K0010) and quantified using the BCA protein quantification assay kit (Beyotime, P0010). Samples were boiled for 5 min at 95°C with the loading buffer (Affinibody LifeScience, AIWB‐0025), and then subjected to sodium dodecyl–sulfate polyacrylamide gel electrophoresis. After gel transfer to membrane filters, primary and secondary antibodies were applied before chemiluminescence detection.

### 4.17 Immunofluorescence

Slides were incubated with primary antibodies, followed by incubation with fluorescent secondary antibodies labeled with Alexa Fluor 488 (Invitrogen, A‐21206) or Alexa Fluor 568 (Invitrogen, A‐10042) for 1.5 h. Slides were then stained with 4′,6‐diamidino‐2‐phenylindole (DAPI) (Beyotime, P0131) and mounted using ProLong Diamond antifade mountant (Thermo Fisher Scientific, P36970). Images were obtained using a Zeiss LSM 880 confocal microscope.

### 4.18 Quantitative Real‐Time Polymerase Chain Reaction (PCR)

Total RNA was extracted from cells using the AxyPrep Multisource Total RNA Miniprep Kit (AXYGEN, AXK‐AP‐MN‐MS‐RNA‐250G). RNA was reverse transcribed into complementary DNA (cDNA) using the Reverse Transcriptase Kit (Takara, RR036A). Quantitative real‐time PCR (qPCR) was then performed in triplicate using the LightCycler 480 (Roche) and qPCR Master Mix (Promega, LS2062). Relative expression of target genes was normalized to GAPDH levels. Sequences of applied qPCR primers were listed in Table S4.

### 4.19 Single‐Cell RNA Sequencing Analysis

Public data were obtained from the NCBI GEO datasets with the accession numbers GSE140228, GSE166635, and GSE138709 (including GSM4116582 and GSM4116586). The Seurat (v4.0.1) R package was employed to perform single‐cell RNA sequencing (scRNA‐Seq) analysis. Briefly, quantifications derived from Cell Ranger were standardized and log2‐transformed using the normalized data functionality within the Seurat package to ensure unique representation and facilitate downstream analysis. The datasets were integrated, followed by running UMAP analysis to visualize the principal components in a low‐dimensional space. UMAP clustering was performed with a resolution parameter set to 0.1.

Cell clusters were identified using the Seurat's FindClusters function. Finally, cell types were automatically annotated by leveraging the SingleR algorithm. Differential gene expression was analyzed using the Seurat's FindMarkers function. KCs and monocyte‐derived macrophages were identified and distinguished based on their established marker gene profiles. Specifically, resident KCs were defined by high expression of CLEC4F, CLEC4A, TIMD4, TIMD3, CLEC4D, CLEC4E, CLEC4A, and VSIG4. Monocyte‐derived macrophages were identified by expression of LY6C, CCR2, ITGAM, GPNMB, CX3CR1, CD206, and TREM2. Immune inhibitory genes were presented in Table S5.

### 4.20 Proteomic Analysis

EV cargo proteins were identified by quantitative proteomic analysis according to procedures described in a previous study [69]. Raw proteomic data are available from the corresponding author upon reasonable request.

### 4.21 ACLY Activity Assay

The ACLY enzymatic activity was assessed using the CheKine Micro ATP Citrate Lyase Activity Assay Kit (Abbkine, KTB1252) according to the manufacturer's instructions. Briefly, isolated cells were homogenized in on ice, centrifuged at 12 000 × *g* for 10 min at 4°C, and the supernatant was used for activity detection. Mechanistically, ACLY activity was quantified by several enzymatic reactions. ACLY catalyzed the cleavage of citrate into acetyl‐CoA and oxaloacetate in the presence of ATP and coenzyme A. The oxaloacetate was then reduced by malate dehydrogenase using NADH, generating malate and NAD^+^. The consumption of NADH in this reaction was proportional to ACLY activity and could be measured by the decrease in absorbance at 340 nm.

### 4.22 Animal Study

Female C57BL/6J and SCID mice at 6–8 weeks were purchased from Zhuhai BesTest Bio‐Tech Co., Ltd. All animal experiments were approved by the Sun Yat‐sen University Institutional Animal Care and Use Committee with the approval numbers 2021002046 and 2021001739. Animals were housed under specific pathogen‐free (SPF) conditions and handled by trained personnel using optimized techniques to reduce animal stress. At the experimental end point, mice were anesthetized with isoflurane and subsequently sacrificed by cervical dislocation prior to tissue collection.

For subcutaneous transplantation model, 5 million Hepa1‐6 murine or SNU449/HepG2 human HCC cells were subcutaneously inoculated into female C57BL/6J or SCID mice, which were then treated with EVs harvested from HCC cells or biomimetic LVs via tail vein injection twice a week until the experimental endpoint. For macrophage depletion, each mice were treated with 150 µL clodronate liposomes (Liposoma B.V., CP‐005‐005) via tail vein injection. Tumor growth was monitored by measuring tumor volume using the following formula: tumor volume = (width^2 × length)/2. To evaluate possible changes in tumor immunity, different immune cell populations in subcutaneous tumors were analyzed by flow cytometry.

For the autochthonous HCC mouse model induced by transposon‐based NRAS and AKT, hydrodynamic transfection was used to induce hepatic expression of myristoylated‐AKT1 and N‐RasV12, leading to HCC tumors formed within 3.5–5 weeks. Specifically, 2 mL saline solution containing 20 µg pT/Caggs‐NRASV12 (Addgene, 20205), 20 µg pT3‐myr‐AKT‐HA (Addgene, 31789), and 2.75 µg pCMV (CAT) T7‐SB100 (Addgene, 34879) was rapidly injected hydrodynamically into the tail vein of mice within 7 s. Intravenous injections of EVs or LVs were started on day 7 after the hydrodynamic transfection and continued twice a week. To evaluate possible changes in tumor immunity, different immune cell populations in autochthonous tumors were analyzed by flow cytometry.

### 4.23 Patient Information

The clinical information of a local HCC patient cohort was included in Table S6. Ethical approval was not required based on regulations of the Ethics Committee of the First Affiliated Hospital, Sun Yat‐sen University, as this study only used anonymized blood samples and archived tumor tissues previously collected for clinical diagnosis with informed written consent from involved patients.

### 4.24 Statistical Analysis

All results are presented as mean ± SD and represent three biological replicates unless otherwise specified. To determine statistical significance between two groups, unpaired Student's *t*‐tests were employed. For comparisons involving more than two groups, one‐way ANOVA was used followed by Tukey's HSD test. Data variance was statistically compared and conformed to be similar. *p*‐value < 0.05 was considered statistically significant. Patient survival was analyzed using the Kaplan–Meier method and statistically compared by the log‐rank *t* test.

## Author Contributions

B.L., Z.L., and D.K. conceived the research. Z.L., H.L., Q.Y., C.Z., Q.C., Y.D., F.C., and Y.Y. conducted the biochemistry, cell biology, and animal experiments. B.J. prepared liposomal vesicles. Z.L., D.K., and B.L. wrote the manuscript and supervised the research.

## Conflicts of Interest

The authors declare no conflicts of interest.

## Supporting information




**Supporting File 1**: advs75345‐sup‐0001‐FigureS1‐S14.pdf.

**Supporting File 2**: advs75345‐sup‐0002‐TableS1‐S6.pdf.

## Data Availability

The data that support the findings of this study are available from the corresponding author upon reasonable request.
