# Supplementary material for: Extracellular Vesicle‐Transferred ATP‐Citrate Lyase Induces Monocyte Differentiation Toward Tumor‐Associated Macrophages and Fuels Hepatocellular Carcinoma Progression
Source: Adv Sci (Weinh). 2026 Apr 17;13(35):e21458. doi: 10.1002/advs.202521458 (PMC13292156; doi:10.1002/advs.202521458)
Supplement: Supplementary file 1 — Supporting File 1: advs75345‐sup‐0001‐FigureS1‐S14.pdf. [file ADVS-13-e21458-s002.pdf]

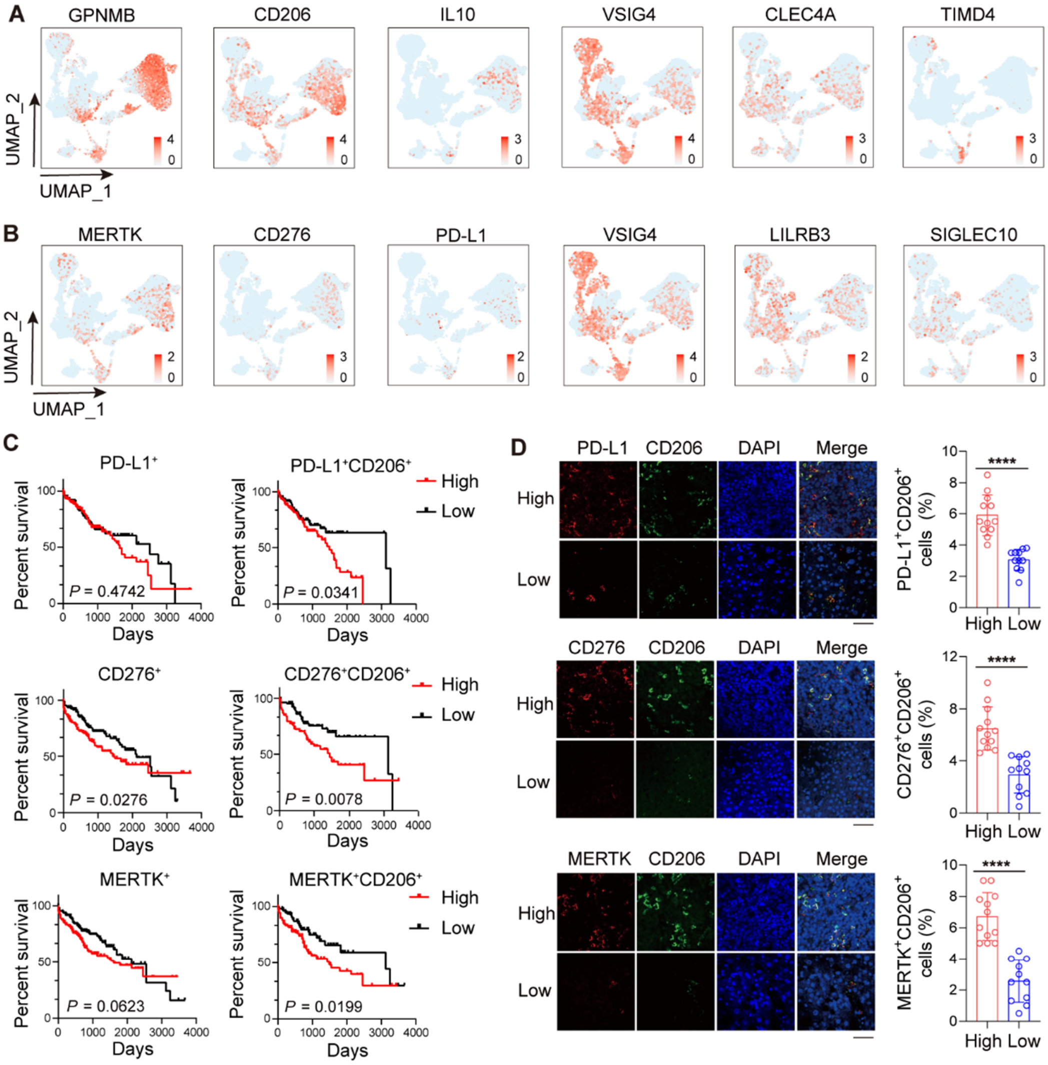
A
GPNMB
CD206
IL10
VSIG4
CLEC4A
TIMD4
UMAP_2
UMAP_1
B
MERTK
CD276
PD-L1
VSIG4
LILRB3
SIGLEC10
C
PD-L1⁺
PD-L1⁺CD206⁺
High
Low
Percent survival
Days
P = 0.4742
P = 0.0341
CD276⁺
CD276⁺CD206⁺
P = 0.0276
P = 0.0078
MERTK⁺
MERTK⁺CD206⁺
P = 0.0623
P = 0.0199
D
PD-L1
CD206
DAPI
Merge
PD-L1⁺CD206⁺ cells (%)
CD276
CD276⁺CD206⁺ cells (%)
MERTK
MERTK⁺CD206⁺ cells (%)
****

Figure S1: Macrophages in HCC tissues harbor immunosuppressive markers.

A. UMAP visualization of macrophage lineage-specific genes in HCC tissues based on scRNA-seq data from GEO datasets (GSE166635, GSE140228, GSE138709).

B. UMAP visualization of immune-inhibitory genes in HCC tissues based on scRNA-seq data from GEO datasets (GSE166635, GSE140228, GSE138709).

C. Kaplan-Meier overall survival analysis of PD-L1^{+}, CD276^{+}, MERTK^{+}, PD-L1^{+}CD206^{+}, CD276^{+}CD206^{+}, and MERTK^{+}CD206^{+} cells in the HCC tumor tissues of TCGA cohort. Data in different patient groups were statistically compared by log-rank t test.

D. Representative immunofluorescence images (left) and quantification (right) of PD-L1, MERTK, CD276, and CD206 in human HCC tissues of a local cohort (n=23, high: n=12, low: n=11). Scale bars represent 50 μm.

Data are presented as mean ± SD. ns, not significant. ****p < 0.0001 (unpaired two-tailed Student's t-test).

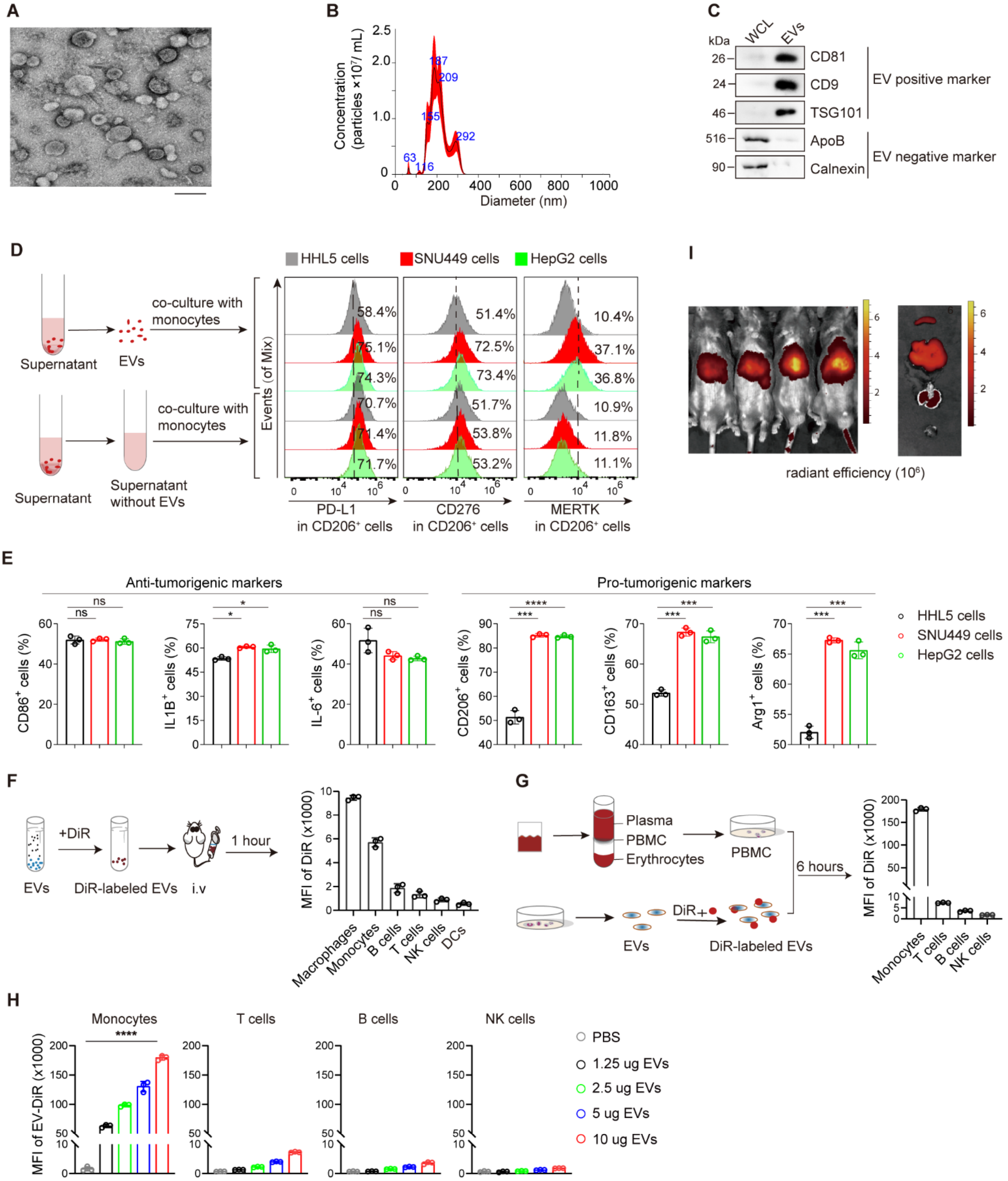
A
B
Concentration (particles ×10^7/ mL)
Diameter (nm)
63
116
155
187
209
292
C
kDa
WCL
EVs
CD81
CD9
TSG101
ApoB
Calnexin
EV positive marker
EV negative marker
D
Supernatant
EVs
co-culture with monocytes
Supernatant
Supernatant without EVs
HHL5 cells
SNU449 cells
HepG2 cells
Events (of Mix)
PD-L1 in CD206+ cells
CD276 in CD206+ cells
MERTK in CD206+ cells
58.4%
75.1%
74.3%
70.7%
71.4%
71.7%
51.4%
72.5%
73.4%
51.7%
53.8%
53.2%
10.4%
37.1%
36.8%
10.9%
11.8%
11.1%
I
radiant efficiency (10^6)
E
Anti-tumorigenic markers
Pro-tumorigenic markers
CD86+ cells (%)
IL1B+ cells (%)
IL-6+ cells (%)
CD206+ cells (%)
CD163+ cells (%)
Arg1+ cells (%)
HHL5 cells
SNU449 cells
HepG2 cells
F
+DiR
EVs
DiR-labeled EVs
i.v
1 hour
MFI of DiR (x1000)
Macrophages
Monocytes
B cells
T cells
NK cells
DCs
G
Plasma
PBMC
Erythrocytes
PBMC
EVs
DiR+
DiR-labeled EVs
6 hours
MFI of DiR (x1000)
Monocytes
T cells
B cells
NK cells
H
Monocytes
T cells
B cells
NK cells
MFI of EV-DiR (x1000)
PBS
1.25 ug EVs
2.5 ug EVs
5 ug EVs
10 ug EVs

Figure S2: ACLY transferred by HCC-derived EV- promotes the differentiation of monocytes into immunosuppressive TAMs.

A. Representation image of transmission electron microscopy (TEM) analysis of EVs secreted from HepG2 cells. Scale bar represents 100 nm.

B. Nanoparticle tracking analysis (NTA) demonstrating the diameter distribution and purity of EVs for DiR labeling. Representative of n = 3 independent experiments.

C. Protein expression of the positive EV markers CD81, TSG101, CD9, and the negative EV markers ApoB and Calnexin in EVs and whole cell lysate (WCL) of HepG2 cells. Representative of n = 3 independent experiments.

D. Representative histogram of flow cytometry analysis in monocytes co-cultured with equal amounts of EVs or EV-depleted medium collected from HHL5, HepG2, or SNU449 cells. Representative of n = 3 independent experiments.

E. Representative expression of indicated markers in monocytes co-cultured with equal amounts of EVs collected from HHL5, HepG2, or SNU449 cells (n=3).

F. Uptake efficiencies of intravenously (i.v.) injected, 10 μg DiR-labeled EVs by different immune cells harvested from mouse peripheral blood were quantified by flow cytometry with the unit of mean fluorescence intensity (MFI) (n=3).

G. Uptake efficiencies of 5 μg DiR-labeled EVs by indicated immune cells from primary PBMCs were quantified by flow cytometry (n=3).

H. Uptake efficiencies of different amounts of DiR-labeled EVs (1.25-10 μg) by different immune cells from primary PBMCs were quantified by flow cytometry (n=3).

I. In vivo fluorescent imaging showing that i.v. injected EVs mainly target liver in mice. Representative of n = 3 independent experiments.

Data are presented as mean ± SD and represent three biological replicates. ns, not significant. ****$p < 0.0001$ (one-way ANOVA with Tukey's HSD test).

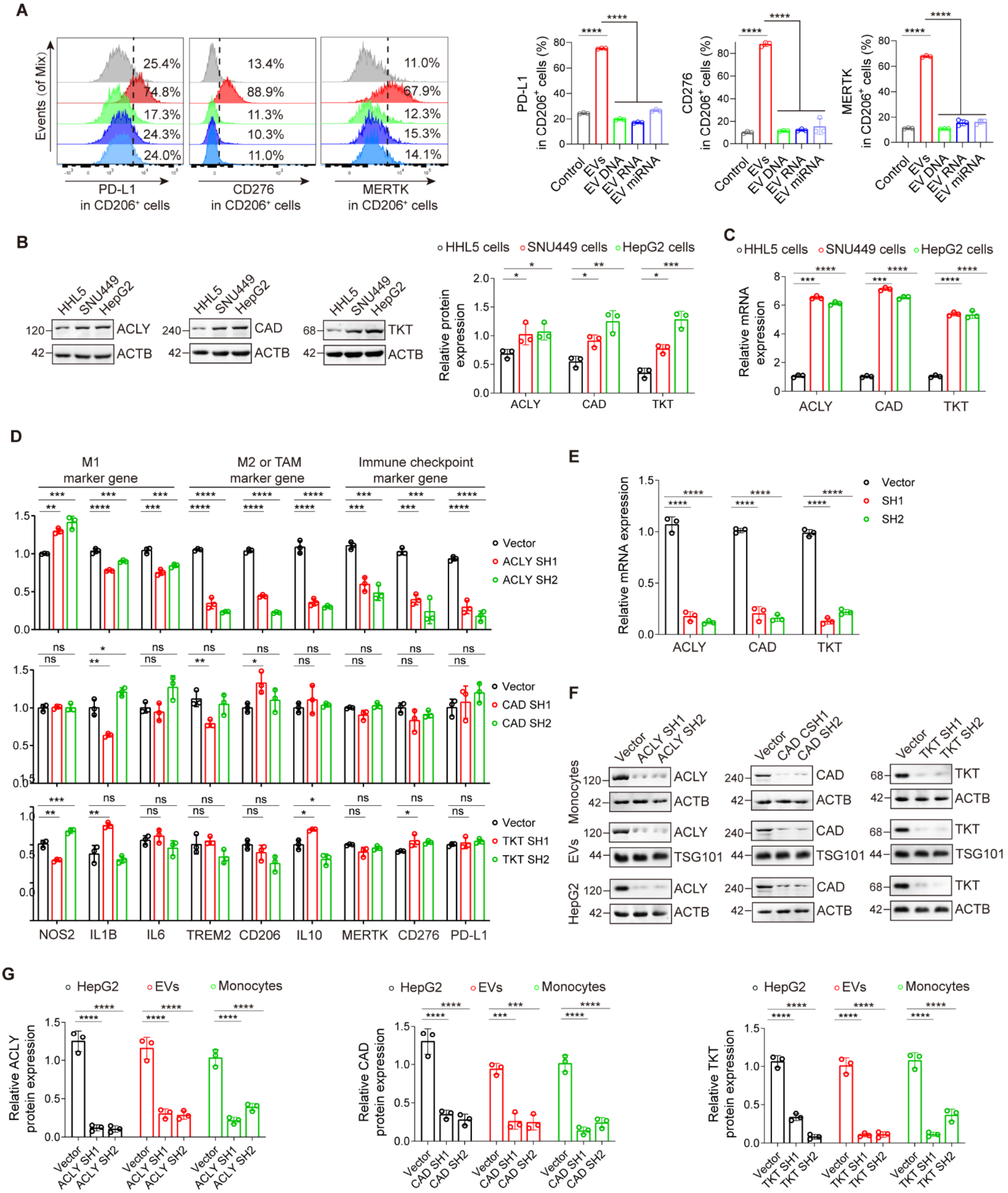
A
Events (of Mix)
25.4%
74.8%
17.3%
24.3%
24.0%
13.4%
88.9%
11.3%
10.3%
11.0%
11.0%
67.9%
12.3%
15.3%
14.1%
PD-L1 in CD206⁺ cells
CD276 in CD206⁺ cells
MERTK in CD206⁺ cells
PD-L1 in CD206⁺ cells (%)
CD276 in CD206⁺ cells (%)
MERTK in CD206⁺ cells (%)
Control
EVs
EV DNA
EV RNA
EV miRNA
B
HHL5
SNU449
HepG2
ACLY
CAD
TKT
ACTB
HHL5 cells
SNU449 cells
HepG2 cells
Relative protein expression
C
Relative mRNA expression
D
M1 marker gene
M2 or TAM marker gene
Immune checkpoint marker gene
Vector
ACLY SH1
ACLY SH2
CAD SH1
CAD SH2
TKT SH1
TKT SH2
NOS2
IL1B
IL6
TREM2
CD206
IL10
MERTK
CD276
PD-L1
E
Relative mRNA expression
Vector
SH1
SH2
F
Monocytes
EVs
HepG2
CAD CSH1
TSG101
G
HepG2
EVs
Monocytes
Relative ACLY protein expression
Relative CAD protein expression
Relative TKT protein expression

Figure S3: EV-containing ACLY proteins promote the shift of monocytes into TAMs.

A. Representative histograms and quantification of PD-L1, CD276, and MERTK levels in $CD206^{+}$ cells were analyzed after the electroporation of nucleic acids (DNA or RNA) purified from HCC-derived EVs. Cells co-cultured with HCC-derived EVs were used as positive control (n=3).

B. Indicated protein levels in HHL5, HepG2, or SNU449 cells. Representative of n = 3 independent experiments.

C. Relative mRNA levels of indicated genes in HHL5, HepG2, or SNU449 cells (n=3).

D. Relative mRNA levels of indicated genes in monocytes incubated with 5 μg EVs secreted from HepG2 cells lentivirally transfected with vector control or two independent shRNAs respectively depleting ACLY, CAD, or TKT for 4 days (n=3).

E. Relative mRNA levels of indicated genes in HepG2 cells (n=3).

F-G. Indicated protein levels in monocytes, EVs and HepG2 cells (n=3).

Data are presented as mean ± SD and represent three biological replicates. ns, not significant. *p < 0.05, **p < 0.01, ***p < 0.001, ****p < 0.0001 (one-way ANOVA with Tukey's HSD test).

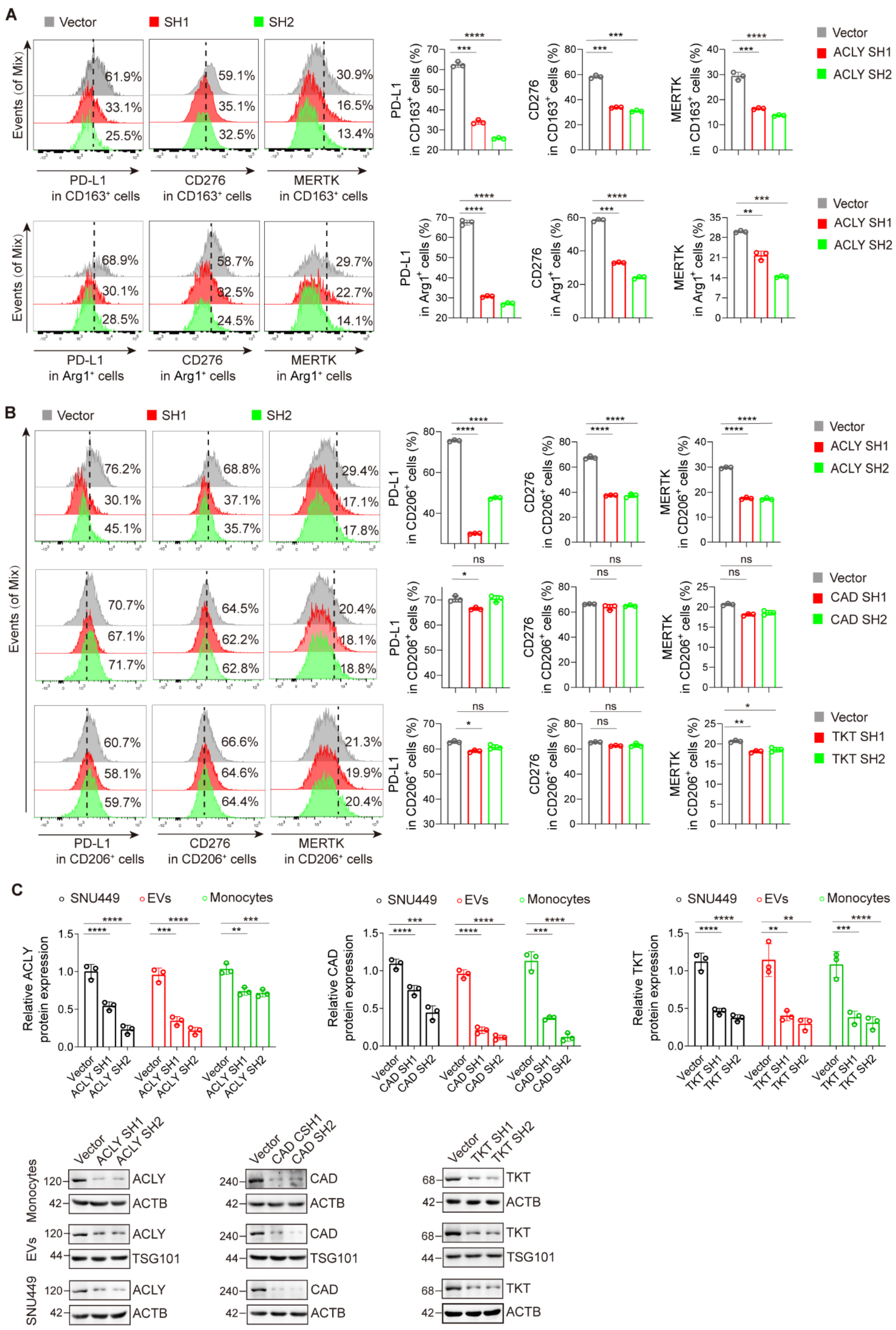

A
B
C
Vector
SH1
SH2
ACLY SH1
ACLY SH2
CAD SH1
CAD SH2
TKT SH1
TKT SH2
Events (of Mix)
PD-L1 in CD163⁺ cells
CD276 in CD163⁺ cells
MERTK in CD163⁺ cells
PD-L1 in Arg1⁺ cells
CD276 in Arg1⁺ cells
MERTK in Arg1⁺ cells
PD-L1 in CD206⁺ cells
CD276 in CD206⁺ cells
MERTK in CD206⁺ cells
SNU449
EVs
Monocytes
Relative ACLY protein expression
Relative CAD protein expression
Relative TKT protein expression
ACLY
ACTB
CAD
TKT
TSG101

Figure S4: EV-containing ACLY proteins promote the shift of monocytes into TAMs.

A. Representative histograms (left) and quantifications (right) of indicated markers in monocytes incubated with 5 μg EVs secreted by HepG2 cells lentivirally transfected with vector control or two independent shRNAs respectively depleting ACLY, CAD, or TKT (n=3).

B. Representative histograms (left) and quantifications (right) of indicated markers in monocytes incubated with 5 μg EVs secreted by SNU449 cells lentivirally transfected with vector control or two independent shRNAs respectively depleting ACLY, CAD, or TKT (n=3).

C. Indicated protein levels in monocytes, EVs and SNU449 cells (n=3).

Data are presented as mean ± SD and represent three biological replicates. ns, not significant. *p < 0.05, **p < 0.01, ***p < 0.001, ****p < 0.0001 (one-way ANOVA with Tukey's HSD test).

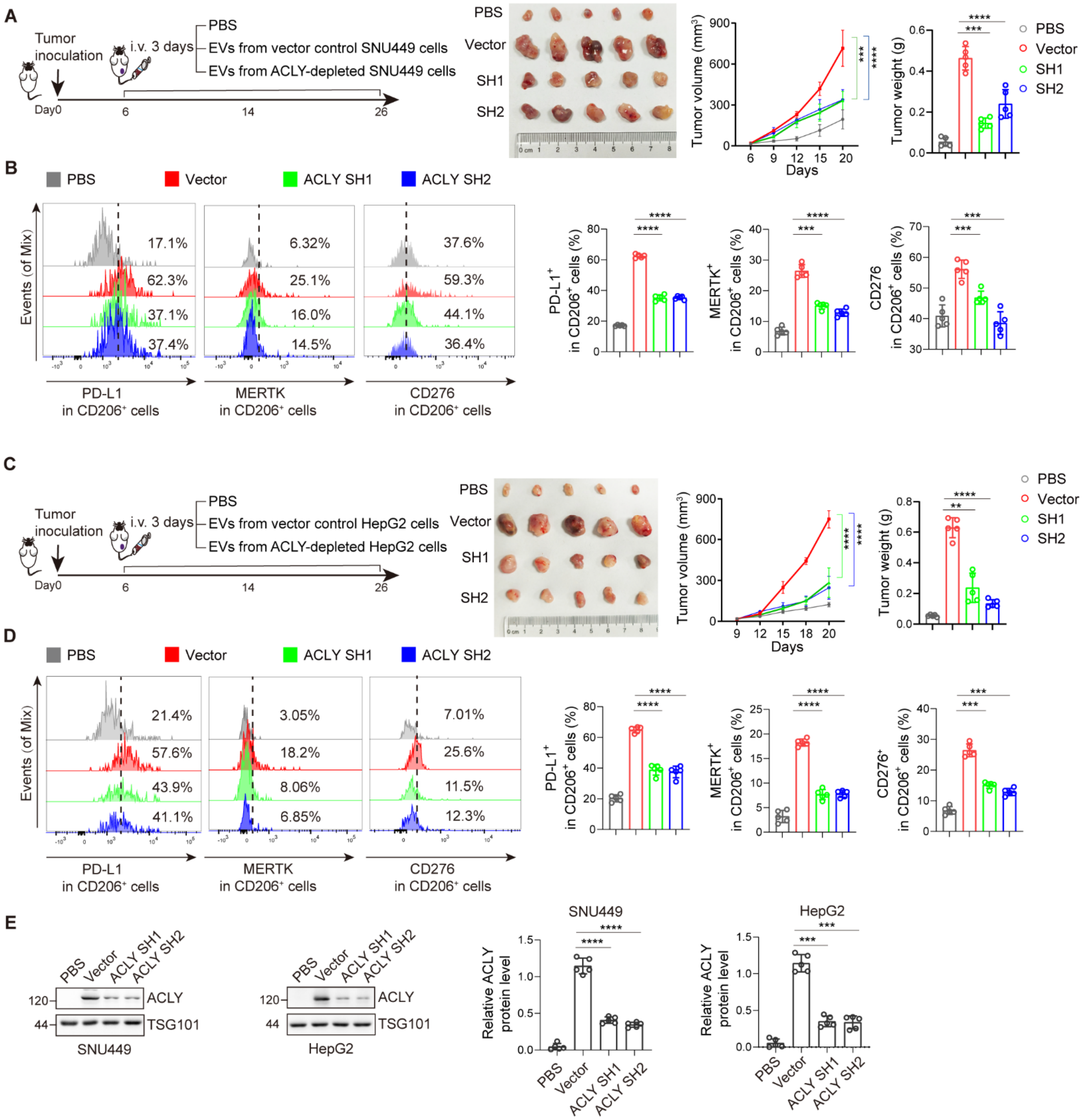
A
Tumor inoculation
i.v. 3 days
PBS
EVs from vector control SNU449 cells
EVs from ACLY-depleted SNU449 cells
Day0
6
14
26
PBS
Vector
SH1
SH2
Tumor volume (mm3)
Days
Tumor weight (g)
B
PBS
Vector
ACLY SH1
ACLY SH2
Events (of Mix)
17.1%
62.3%
37.1%
37.4%
6.32%
25.1%
16.0%
14.5%
37.6%
59.3%
44.1%
36.4%
PD-L1 in CD206+ cells
MERTK in CD206+ cells
CD276 in CD206+ cells
PD-L1+ in CD206+ cells (%)
MERTK+ in CD206+ cells (%)
CD276 in CD206+ cells (%)
C
Tumor inoculation
i.v. 3 days
PBS
EVs from vector control HepG2 cells
EVs from ACLY-depleted HepG2 cells
Day0
6
14
26
PBS
Vector
SH1
SH2
Tumor volume (mm3)
Days
Tumor weight (g)
D
PBS
Vector
ACLY SH1
ACLY SH2
Events (of Mix)
21.4%
57.6%
43.9%
41.1%
3.05%
18.2%
8.06%
6.85%
7.01%
25.6%
11.5%
12.3%
PD-L1 in CD206+ cells
MERTK in CD206+ cells
CD276 in CD206+ cells
PD-L1+ in CD206+ cells (%)
MERTK+ in CD206+ cells (%)
CD276+ in CD206+ cells (%)
E
PBS
Vector
ACLY SH1
ACLY SH2
120
44
ACLY
TSG101
SNU449
HepG2
Relative ACLY protein level

Figure S5: EV-containing ACLY proteins promote the shift of monocytes into TAMs.

A. Flowchart illustrating the experimental design (left), images and growth (middle), and weights (right) of subcutaneous tumors i.v. treated with PBS or 10 μg EVs derived from SNU449 cells transfected with vector control or shRNAs depleting ACLY (n=5).

B. Flow cytometry analysis of immunosuppressive TAMs in subcutaneous tumors as indicated in (A). Left: representative histograms. Right: quantification of histograms (n=5).

C. Flowchart illustrating the experimental design (left), images and growth (middle), and weights (right) of subcutaneous tumors i.v. treated with PBS or 10 μg EVs derived from HepG2 cells transfected with vector control or shRNAs depleting ACLY (n=5).

D. Flow cytometry analysis of immunosuppressive TAMs in subcutaneous tumors as indicated in (C). Left: representative histograms. Right: quantification of histograms (n=5).

E. Protein levels of ACLY in EVs derived of SNU449 (A) or HepG2 (C) cells (n=5).

Data are presented as mean ± SD. ns, not significant. *p < 0.05, **p < 0.01, ***p < 0.001, ****p < 0.0001 (one-way ANOVA with Tukey's HSD test).

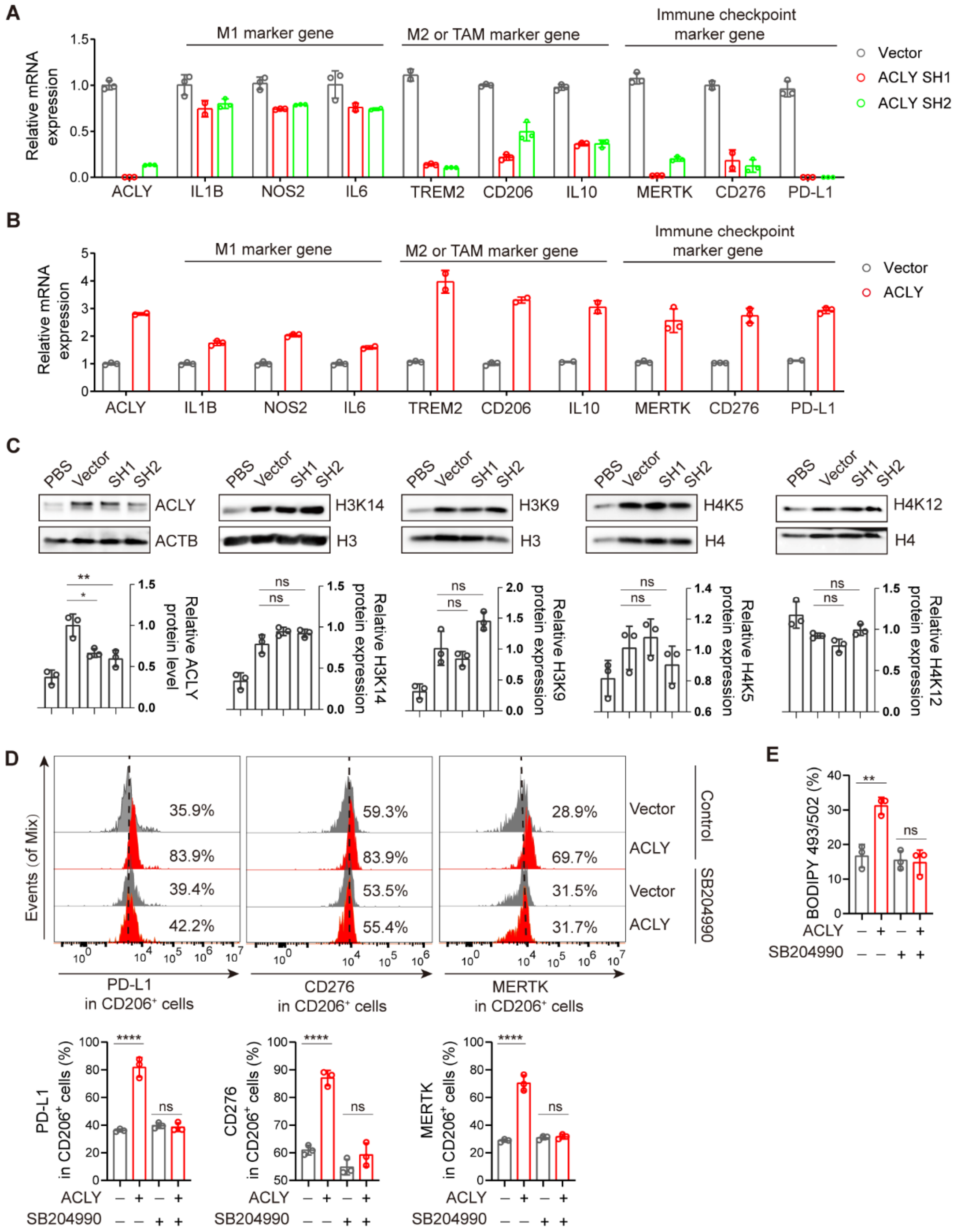
A
M1 marker gene
M2 or TAM marker gene
Immune checkpoint marker gene
Relative mRNA expression
Vector
ACLY SH1
ACLY SH2
ACLY
IL1B
NOS2
IL6
TREM2
CD206
IL10
MERTK
CD276
PD-L1
B
Vector
ACLY
C
PBS
Vector
SH1
SH2
ACLY
ACTB
H3K14
H3
H3K9
H4K5
H4
H4K12
Relative ACLY protein level
Relative H3K14 protein expression
Relative H3K9 protein expression
Relative H4K5 protein expression
Relative H4K12 protein expression
D
Events (of Mix)
35.9%
83.9%
39.4%
42.2%
59.3%
83.9%
53.5%
55.4%
28.9%
69.7%
31.5%
31.7%
Vector
ACLY
Control
SB204990
PD-L1 in CD206⁺ cells
CD276 in CD206⁺ cells
MERTK in CD206⁺ cells
PD-L1 in CD206⁺ cells (%)
CD276 in CD206⁺ cells (%)
MERTK in CD206⁺ cells (%)
ACLY
SB204990
E
BODIPY 493/502 (%)

Figure S6: EV-enriched ACLY induces immunosuppressive TAMs through reprogramming fatty acid metabolism.

A-B. Relative mRNA levels of indicated genes in monocytes following direct silencing (A) or overexpression (B) of ACLY (n=3).

C. Levels of indicated histone acetylation in monocytes incubated with or without 5 ug EVs secreted from HepG2 cells with or without ACLY depletion for 4 days (n=3).

D. Representative histograms (upper panel) and quantification (lower panel) of indicated markers in monocytes treated with 5 μg EVs secreted from HepG2 cells expressing vector control or ACLY for 4 days, in the presence or absence of 20 μM SB204990 (n=3).

E. Quantification of BODIPY 493/503 staining in monocytes indicated in (D) (n=3).

Data are presented as mean ± SD and represent three biological replicates. ns, not significant. *$p < 0.05$, **$p < 0.01$, ***$p < 0.001$, ****$p < 0.0001$ (one-way ANOVA with Tukey's HSD test).

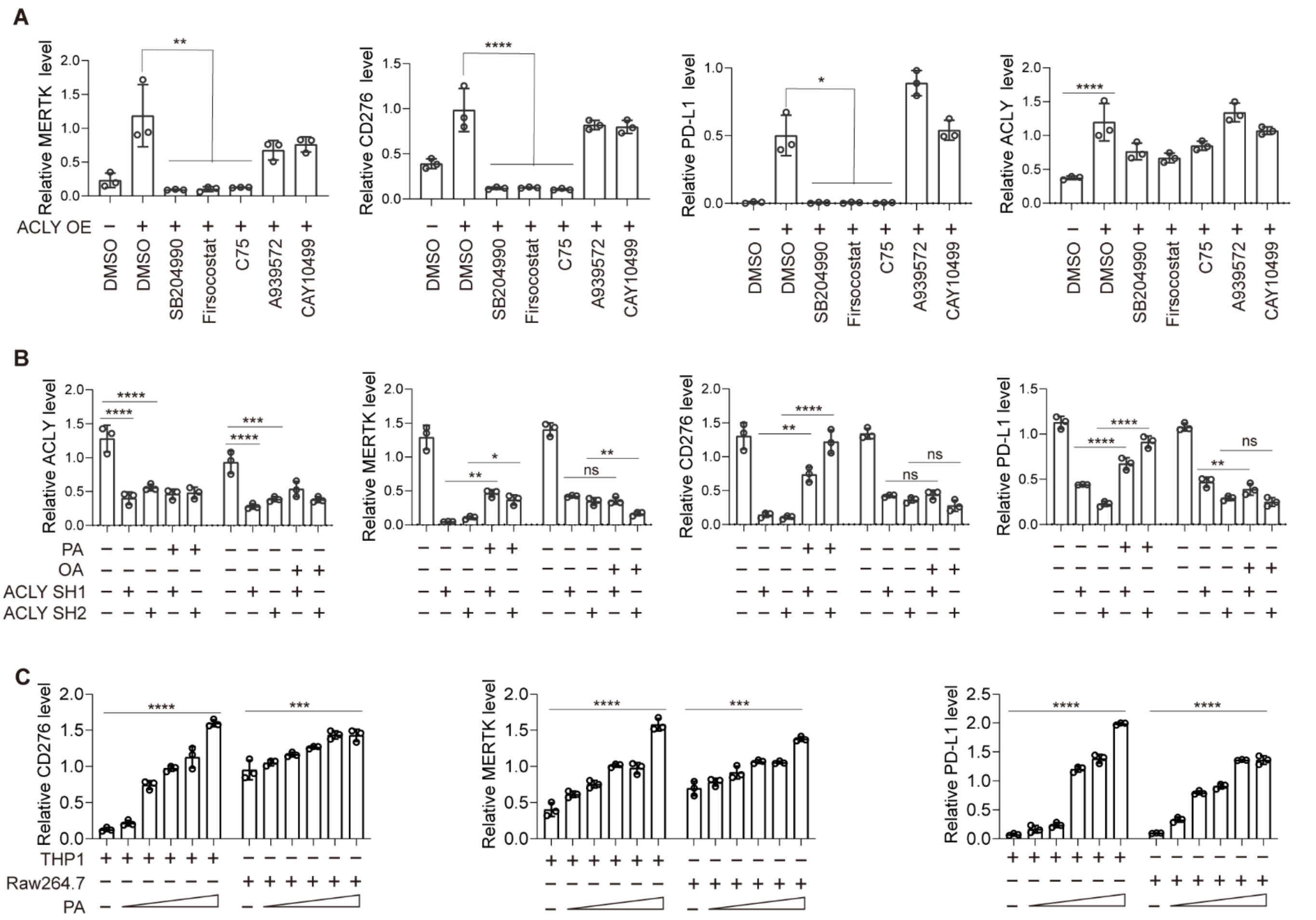
A
Relative MERTK level
Relative CD276 level
Relative PD-L1 level
Relative ACLY level
ACLY OE
DMSO
SB204990
Firsocostat
C75
A939572
CAY10499
B
Relative ACLY level
Relative MERTK level
Relative CD276 level
Relative PD-L1 level
PA
OA
ACLY SH1
ACLY SH2
ns
C
Relative CD276 level
Relative MERTK level
Relative PD-L1 level
THP1
Raw264.7
PA

Figure S7: ACLY-encapsulated EVs drive TAM polarization by producing palmitate.

A. Quantitative protein levels of indicated immunosuppressive markers in THP1 cells incubated with 5 μg of EVs secreted by HepG2 cells lentivirally transfected with vector control or ACLY for 2 days, followed by treatment with different inhibitors for another 2 days related to Figure 4G (n=3).

B. Quantitative protein levels of indicated proteins in THP1 cells incubated with 5 μg EVs derived from HepG2 cells with or without ACLY depletion for 4 days, followed by treatment with 0.5 mM palmitic acid (PA) or oleic acid (OA) for 8 hours related to Figure 4H and 4I (n=3).

C. Quantitative protein levels of indicated immunosuppressive markers in THP1 and Raw264.7 cells treated without or with increasing doses of PA (50-500 μM) for 8 hours related to Figure 4L (n=3).

Data are presented as mean ± SD and represent three biological replicates. ns, not significant. *p < 0.05, **p < 0.01, ***p < 0.001, ****p < 0.0001 (one-way ANOVA with Tukey's HSD test).

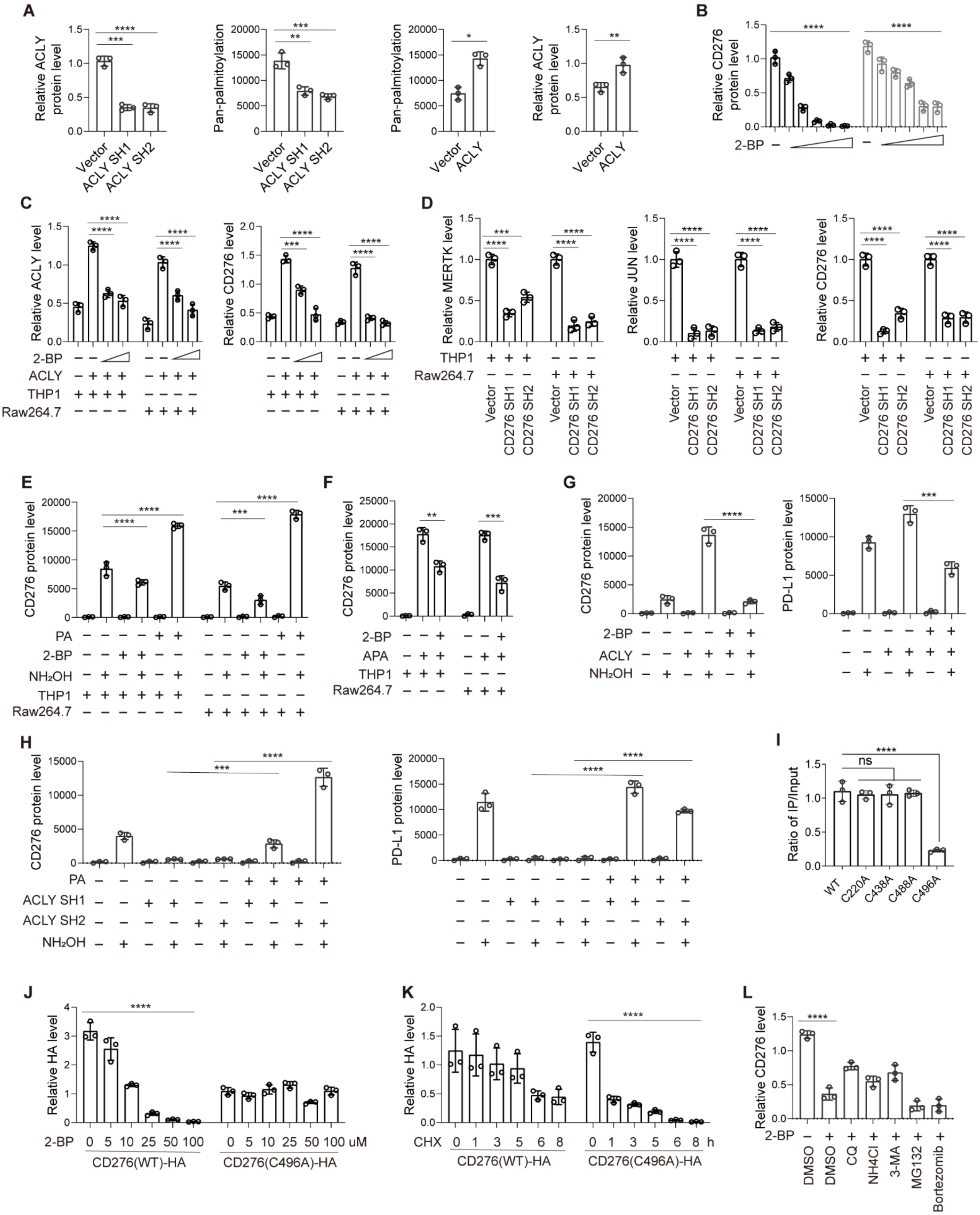

A
Relative ACLY protein level
Pan-palmitoylation
Vector
ACLY SH1
ACLY SH2
ACLY
B
Relative CD276 protein level
2-BP
C
Relative ACLY level
Relative CD276 level
2-BP
ACLY
THP1
Raw264.7
D
Relative MERTK level
Relative JUN level
Relative CD276 level
THP1
Raw264.7
Vector
CD276 SH1
CD276 SH2
E
CD276 protein level
PA
2-BP
NH₂OH
THP1
Raw264.7
F
CD276 protein level
2-BP
APA
THP1
Raw264.7
G
CD276 protein level
PD-L1 protein level
2-BP
ACLY
NH₂OH
H
CD276 protein level
PD-L1 protein level
PA
ACLY SH1
ACLY SH2
NH₂OH
I
Ratio of IP/Input
ns
WT
C220A
C438A
C488A
C496A
J
Relative HA level
2-BP 0 5 10 25 50 100 0 5 10 25 50 100 uM
CD276(WT)-HA
CD276(C496A)-HA
K
Relative HA level
CHX 0 1 3 5 6 8 0 1 3 5 6 8 h
CD276(WT)-HA
CD276(C496A)-HA
L
Relative CD276 level
2-BP
DMSO
DMSO
CQ
NH4Cl
3-MA
MG132
Bortezomib

Figure S8: EV-enriched ACLY drives palmitoylation of immune checkpoint proteins.

A. Quantitative pan-palmitoylation levels in macrophages incubated with 5 μg EVs secreted from HepG2 cells with or without ACLY depletion or overexpression for 4 days related to Figure 5A (n=3). Left, one-way ANOVA with Tukey's HSD test. Right, Two-tailed unpaired Student's t-test.

B. Quantitative levels of CD276 expression in THP1 and Raw264.7 cells treated without or with increasing doses of 2-BP (10-100 μM) for 24 hours related to Figure 5B (n=3).

C. Quantitative levels of CD276 expression in THP1 and Raw264.7 cells incubated with 5 μg EVs derived from HepG2 cells without or with ACLY OE for 4 days, followed by treatment without or with 50-100 μM 2-BP for 24 hours related to Figure 5E (n=3).

D. Quantitative levels of indicated proteins in THP1 and Raw264.7 cells transfected without or with two independent shRNAs depleting CD276 related to Figure 5F (n=3).

E-F. Quantitative palmitoylation levels of CD276 in THP1 cells were detected by the ABE (E) and Click-iT labeling assay (F). For the ABE assay, THP1 cells were treated without or with 100 μM 2-BP or 500 μM PA, in the presence or absence of 0.5 M NH_2OH. For the Click-iT labeling assay, THP1 cells were treated without or with 100 μM 15-azido-pentadecanoic acid (APA) and/or 100 μM 2-BP related to Figure 5H-I (n=3).

G. Quantitative CD276 and PD-L1 palmitoylation in THP1 cells co-culturing with 5 ug EVs derived from HepG2 cells with or without ACLY for 4 days, treated with 100 uM 2-BP for 24 h, and detected by ABE assay related to Figure 5J (n=3).

H. Quantitative CD276 and PD-L1 palmitoylation in THP1 cells co-culturing with 5 ug EVs derived from HepG2 cells with or without ACLY for 4 days, treated with 500 uM PA for 24 h, and detected by ABE assay related to Figure 5K (n=3).

I. Quantitative palmitoylation levels of CD276-WT and indicated mutants in THP1 cells measured by the ABE assay related to Figure 5L (n=3).

J. Quantitative expression of HA-tagged CD276 WT or C496A mutant in THP1 cells treated without or with increasing doses of 2-BP for 24 hours related to Figure 5M (n=3).

K. Quantitative protein levels of exogenous CD276 (WT) and CD276 (C496A)-HA in CHX -chase assay for indicated periods of time related to Figure 5N (n=3).

L. Quantitative protein levels of CD276 in THP1 cells treated with DMSO control or indicated inhibitors for 24 hours related to Figure 5O (n=3).

Data are presented as mean ± SD and represent three biological replicates. ns, not significant. *p < 0.05, **p < 0.01, ***p < 0.001, ****p < 0.0001. One-way ANOVA with Tukey's HSD test.

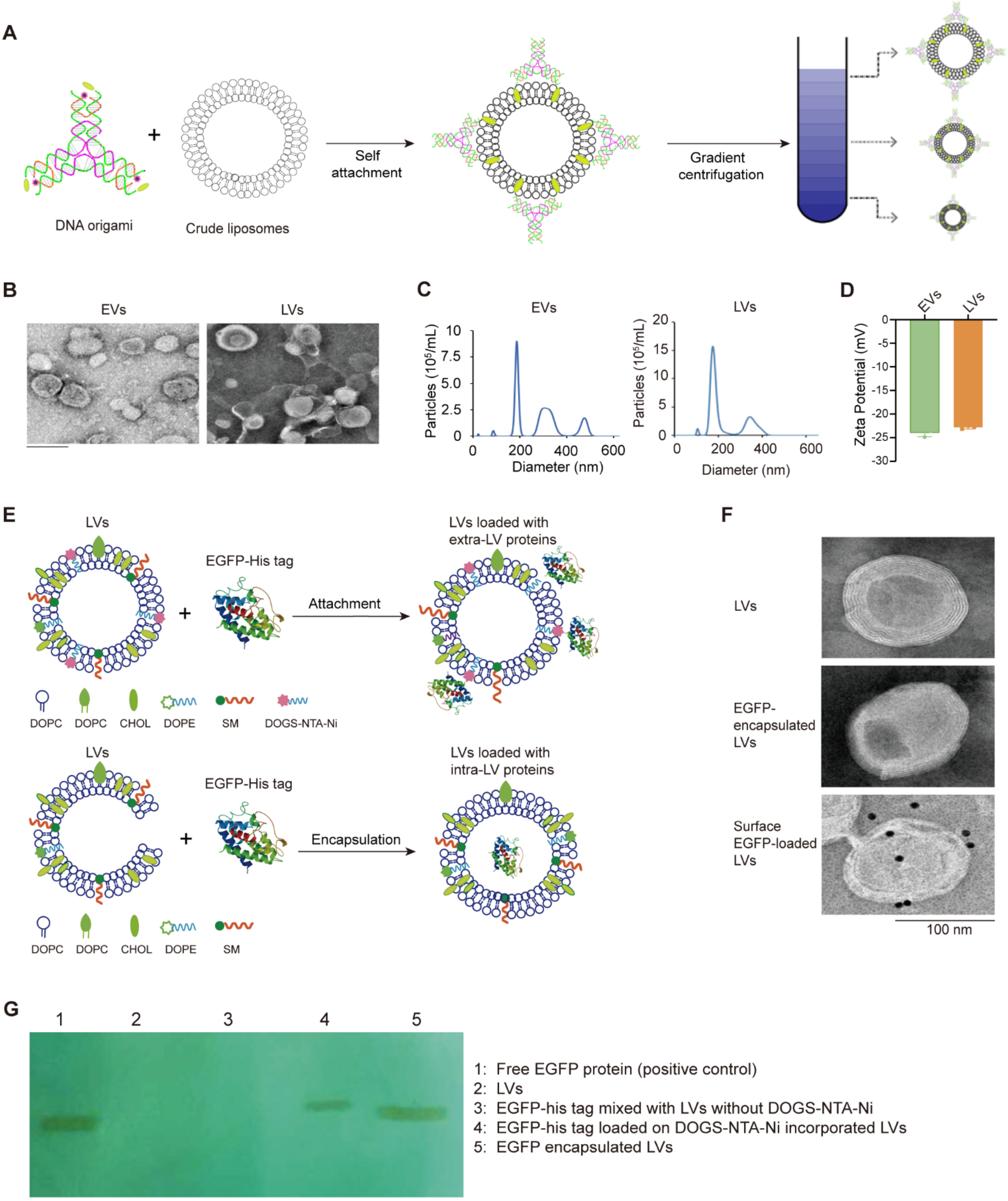
A
DNA origami
Crude liposomes
Self attachment
Gradient centrifugation
B
EVs
LVs
C
EVs
Particles (10⁵/mL)
Diameter (nm)
LVs
D
EVs
LVs
Zeta Potential (mV)
E
LVs
EGFP-His tag
Attachment
LVs loaded with extra-LV proteins
DOPC
DOPC
CHOL
DOPE
SM
DOGS-NTA-Ni
LVs
EGFP-His tag
Encapsulation
LVs loaded with intra-LV proteins
DOPC
DOPC
CHOL
DOPE
SM
F
LVs
EGFP-encapsulated LVs
Surface EGFP-loaded LVs
100 nm
G
1
2
3
4
5
1: Free EGFP protein (positive control)
2: LVs
3: EGFP-his tag mixed with LVs without DOGS-NTA-Ni
4: EGFP-his tag loaded on DOGS-NTA-Ni incorporated LVs
5: EGFP encapsulated LVs

Figure S9: Design of an EV-memetic liposomal system.

A. Schematic diagram of purifying EV-memetic LVs using DNA origami. We employed DNA nanotechnology to construct brick-like DNA origami that can attach to various sizes of crude liposomes. LVs ranging from 30-130 nm were then separated by gradient centrifugation, followed by DNase digestion to remove the DNA origami.

B. Representative TEM pictures of EVs and LVs. Scale bar represents 100 nm.

C. Particle size distribution of EVs and LVs in diameter.

D. Zeta potential analysis of EVs and LVs.

E. Schematic diagram of loading extra- and intra- LV proteins. Incorporation of DOGS-NTA-Ni enabled LVs to bind His-tagged proteins via Ni-His interactions, whereas adding proteins before LV formation allowed their encapsulation within the LV core.

F. Representative pictures of LVs (top), EGFP-encapsulated LVs (middle), and surface EGFP-loaded LVs (bottom) measured by the colloidal gold-labeling immunoelectron microscopy. Scale bar represents 100 nm.

G. Coomassie staining analysis of LVs loaded without or with EGFP as the extra- or intra-LV cargo as indicated.

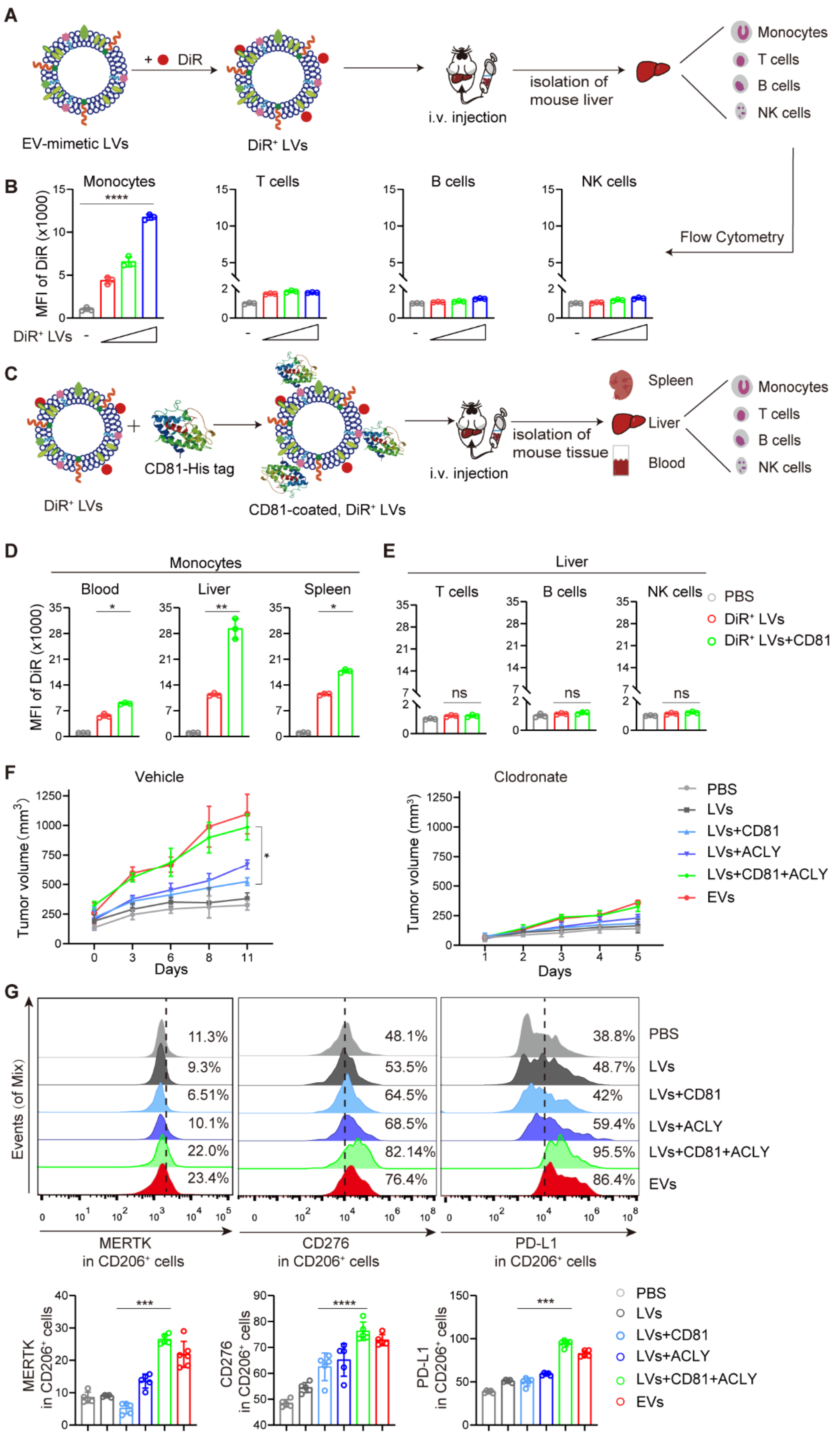
A
DiR
EV-mimetic LVs
DiR⁺ LVs
i.v. injection
isolation of mouse liver
Monocytes
T cells
B cells
NK cells
Flow Cytometry
B
Monocytes
T cells
B cells
NK cells
MFI of DiR (x1000)
DiR⁺ LVs
C
DiR⁺ LVs
CD81-His tag
CD81-coated, DiR⁺ LVs
i.v. injection
isolation of mouse tissue
Spleen
Liver
Blood
D
Monocytes
Blood
Liver
Spleen
E
Liver
T cells
B cells
NK cells
PBS
DiR⁺ LVs
DiR⁺ LVs+CD81
F
Vehicle
Clodronate
Tumor volume (mm³)
Days
PBS
LVs
LVs+CD81
LVs+ACLY
LVs+CD81+ACLY
EVs
G
Events (of Mix)
MERTK in CD206⁺ cells
CD276 in CD206⁺ cells
PD-L1 in CD206⁺ cells
11.3%
9.3%
6.51%
10.1%
22.0%
23.4%
48.1%
53.5%
64.5%
68.5%
82.14%
76.4%
38.8%
48.7%
42%
59.4%
95.5%
86.4%
PBS
LVs
LVs+CD81
LVs+ACLY
LVs+CD81+ACLY
EVs
MERTK in CD206⁺ cells
CD276 in CD206⁺ cells
PD-L1 in CD206⁺ cells

Figure S10: ACLY-encapsulated LVs promote tumor growth through TAM activation.

A-B. Delivery efficiency of various amounts (10-250 μg) of i.v. injected, DiR-labeled naked LVs to different immune cells in mouse liver 1 hour after injection. Upper panel (A): schematic diagram of LV administration. Lower panel (B): Flow cytometry quantification of DiR-positive fluorescence intensity in isolated immune cells (n=3).

C. Schematic diagram showing that i.v. injected, DiR-labeled LVs coated with CD81 fuse to different immune cells in mouse liver and other tissues 1 hour after injection.

D. Delivery efficiency of DiR-labeled LVs coated without or with CD81 to monocytes isolated from mouse peripheral blood, liver, and spleen (n=3).

E. Delivery efficiency of DiR-labeled LVs coated without or with CD81 to various immune cells isolated from mouse liver (n=3).

F. Tumor volumes of Hepa1-6-derived subcutaneous tumors treated with PBS, EVs, or LVs carrying CD81 and/or ACLY via tail vein injection. Macrophage depletion was achieved by co-injection of 200 μL clodronate liposomes per mouse (n=5).

G. Flow cytometry analysis of immunosuppressive TAMs harvested from subcutaneous tumors as indicated in (F) (n=5).

Data are presented as mean ± SD and represent three biological replicates. ns, not significant. *$p < 0.05$, **$p < 0.01$, ***$p < 0.001$, ****$p < 0.0001$ (one-way ANOVA with Tukey’s HSD test).

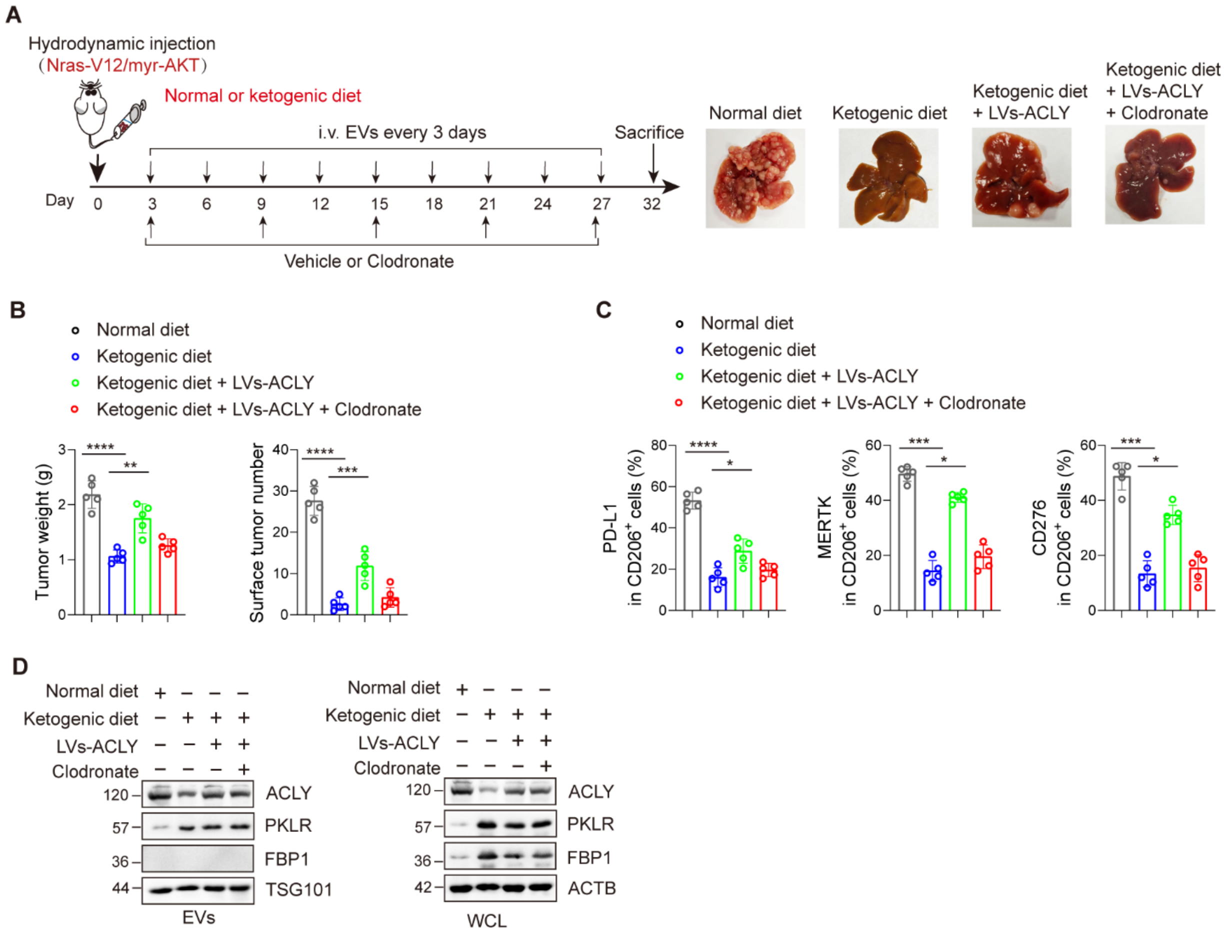
A
Hydrodynamic injection
(Nras-V12/myr-AKT)
Normal or ketogenic diet
i.v. EVs every 3 days
Sacrifice
Day 0 3 6 9 12 15 18 21 24 27 32
Vehicle or Clodronate
Normal diet
Ketogenic diet
Ketogenic diet + LVs-ACLY
Ketogenic diet + LVs-ACLY + Clodronate
B
Normal diet
Ketogenic diet
Ketogenic diet + LVs-ACLY
Ketogenic diet + LVs-ACLY + Clodronate
Tumor weight (g)
Surface tumor number
C
PD-L1 in CD206+ cells (%)
MERTK in CD206+ cells (%)
CD276 in CD206+ cells (%)
D
Normal diet
Ketogenic diet
LVs-ACLY
Clodronate
ACLY
PKLR
FBP1
TSG101
EVs
ACTB
WCL

Figure S11：ACLY-encapsulated LVs promote tumor growth through TAM activation under a ketogenic diet.

A. Schematic diagram (left) and representative livers (right) harvested from the HCC model created by hydrodynamically injected Nras-V12 and myr-AKT, i.v. treated without or with ketogenic diet and/or LVs-ACLY. Macrophage depletion was achieved using clodronate liposomes. LVs-ACLY, LVs carrying ACLY.

B. Weight (left) and surface tumor number (right) of liver tissues harvested from the HCC model created by hydrodynamically injected Nras-V12 and myr-AKT, i.v. treated with or without ketogenic diet and/or LVs-ACLY (n=5).

C. Quantification of flow cytometry analysis of immunosuppressive TAMs isolated from mouse livers indicated in (B) (n=5).

D. Indicated protein levels in mice liver. Representative of n = 3 independent experiments.

Data are presented as mean ± SD and represent three biological replicates. ns, not significant. *p < 0.05, **p < 0.01, ***p < 0.001, ****p < 0.0001 (one-way ANOVA with Tukey's HSD test).

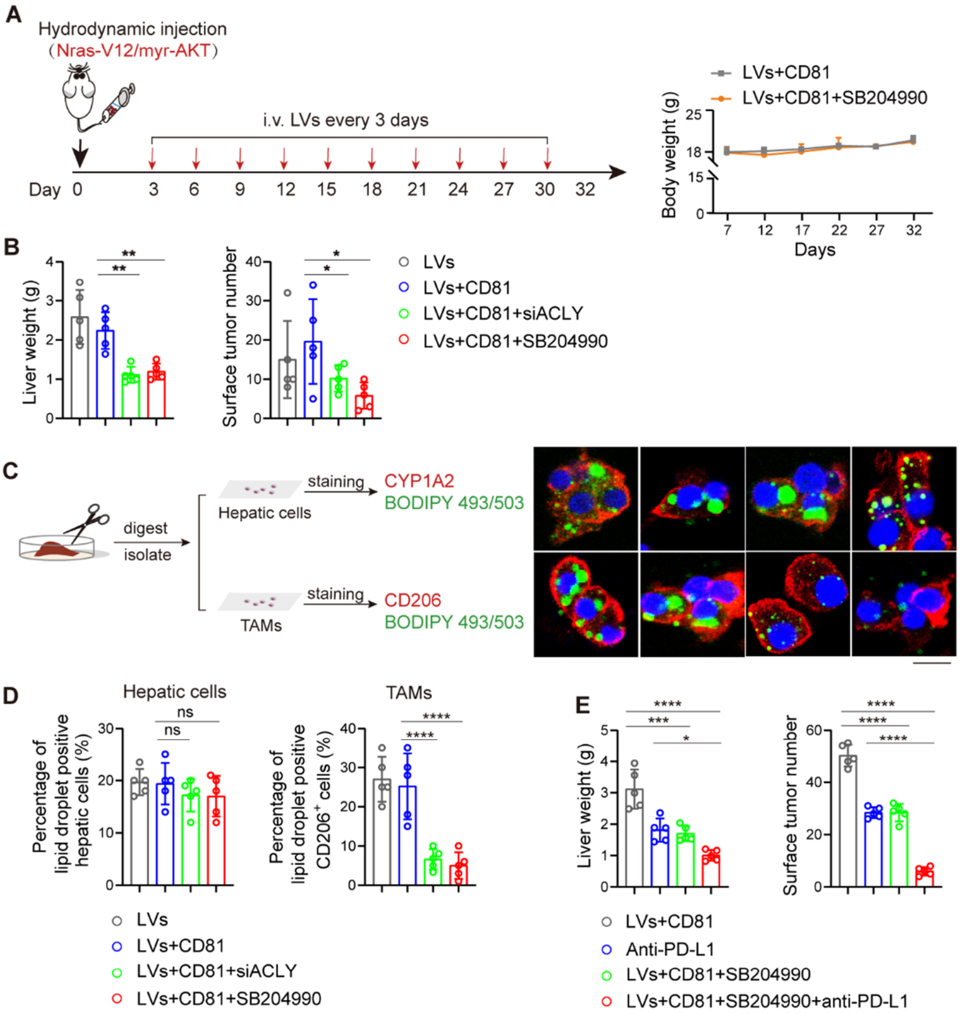
A
Hydrodynamic injection
(Nras-V12/myr-AKT)
i.v. LVs every 3 days
Day 0 3 6 9 12 15 18 21 24 27 30 32
LVs+CD81
LVs+CD81+SB204990
Body weight (g)
Days
B
Liver weight (g)
Surface tumor number
LVs
LVs+CD81
LVs+CD81+siACLY
LVs+CD81+SB204990
C
digest
isolate
staining
Hepatic cells
CYP1A2
BODIPY 493/503
TAMs
CD206
BODIPY 493/503
D
Hepatic cells
Percentage of lipid droplet positive hepatic cells (%)
ns
TAMs
Percentage of lipid droplet positive CD206+ cells (%)
LVs
LVs+CD81
LVs+CD81+siACLY
LVs+CD81+SB204990
E
Liver weight (g)
Surface tumor number
LVs+CD81
Anti-PD-L1
LVs+CD81+SB204990
LVs+CD81+SB204990+anti-PD-L1

Figure S12: LVs loaded with ACLY inhibitors suppress HCC tumor progression.

A. Schematic diagram (left) and body weight (right) of C57BL/6J mice i.v. treated with CD81-coated LVs loaded with or without the ACLY inhibitor SB204990.

B. Weight (left) and surface tumor number (right) of liver tissues harvested from the HCC model created by hydrodynamically injected Nras-V12 and myr-AKT, i.v. treated with or without LVs loaded with the ACLY inhibitor SB204990 or siRNAs depleting ACLY (n=5).

C-D. Representative immunofluorescence images (C) and quantification (D) of BODIPY 493/503 staining to visualize lipid droplets in hepatic cells and macrophages collected from mouse livers as indicated in (B) (n=5). Note that CYP1A2 is a hepatocyte marker. Scale bar represents 10 μm.

E. Weight (left) and surface tumor number (right) of liver tissues harvested from the HCC model created by hydrodynamically injected Nras-V12 and myr-AKT, i.v. treated with or without anti-PD-L1 antibodies and/or CD81-coated LVs loaded without or with SB204990 (n=5).

Data are presented as mean ± SD and represent three biological replicates. ns, not significant. *p < 0.05, **p < 0.01, ***p < 0.001, ****p < 0.0001 (one-way ANOVA with Tukey's HSD test).

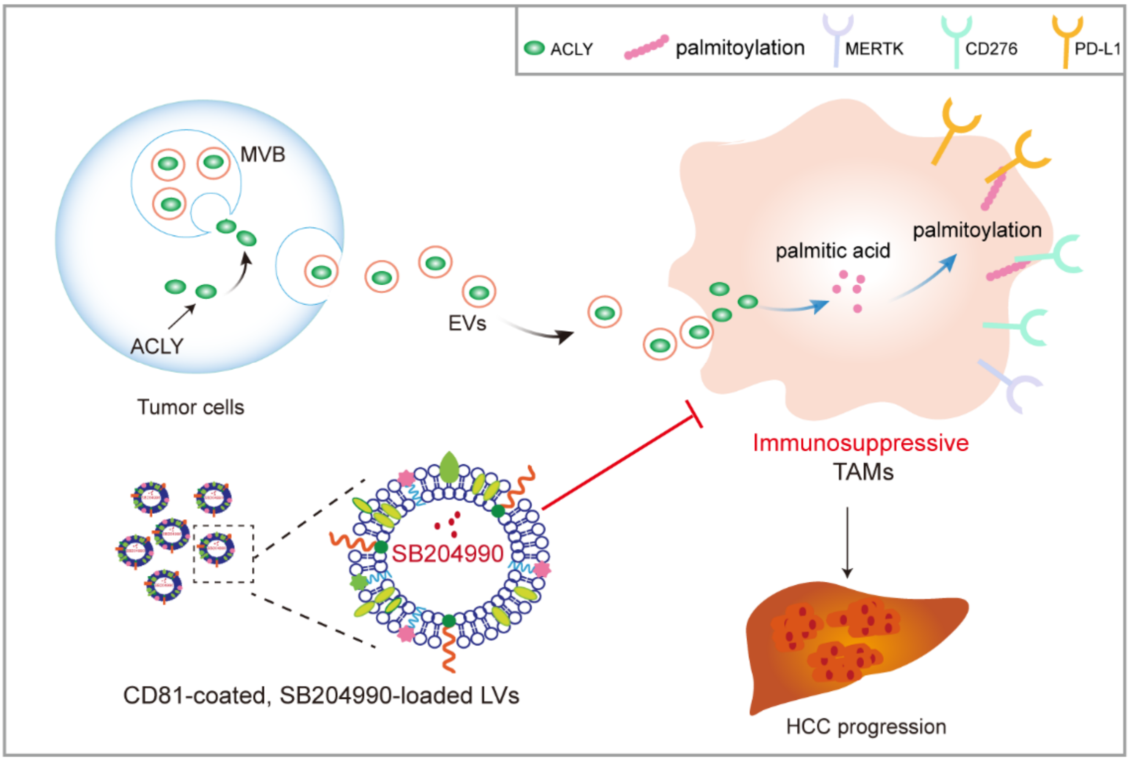

Figure S13: Schematic illustration of theoretical model.

Schematic illustration depicting our proposed model. HCC tumor-derived EVs deliver ACLY to recipient monocytes, enhancing the palmitoylation and stability of immune checkpoint proteins CD276 and PD-L1, therefore leading to the differentiation of immunosuppressive TAMs. In contrast, CD81-coated LVs carrying the ACLY inhibitor SB204990 suppress TAM differentiation and enhance immunotherapeutic efficacy.

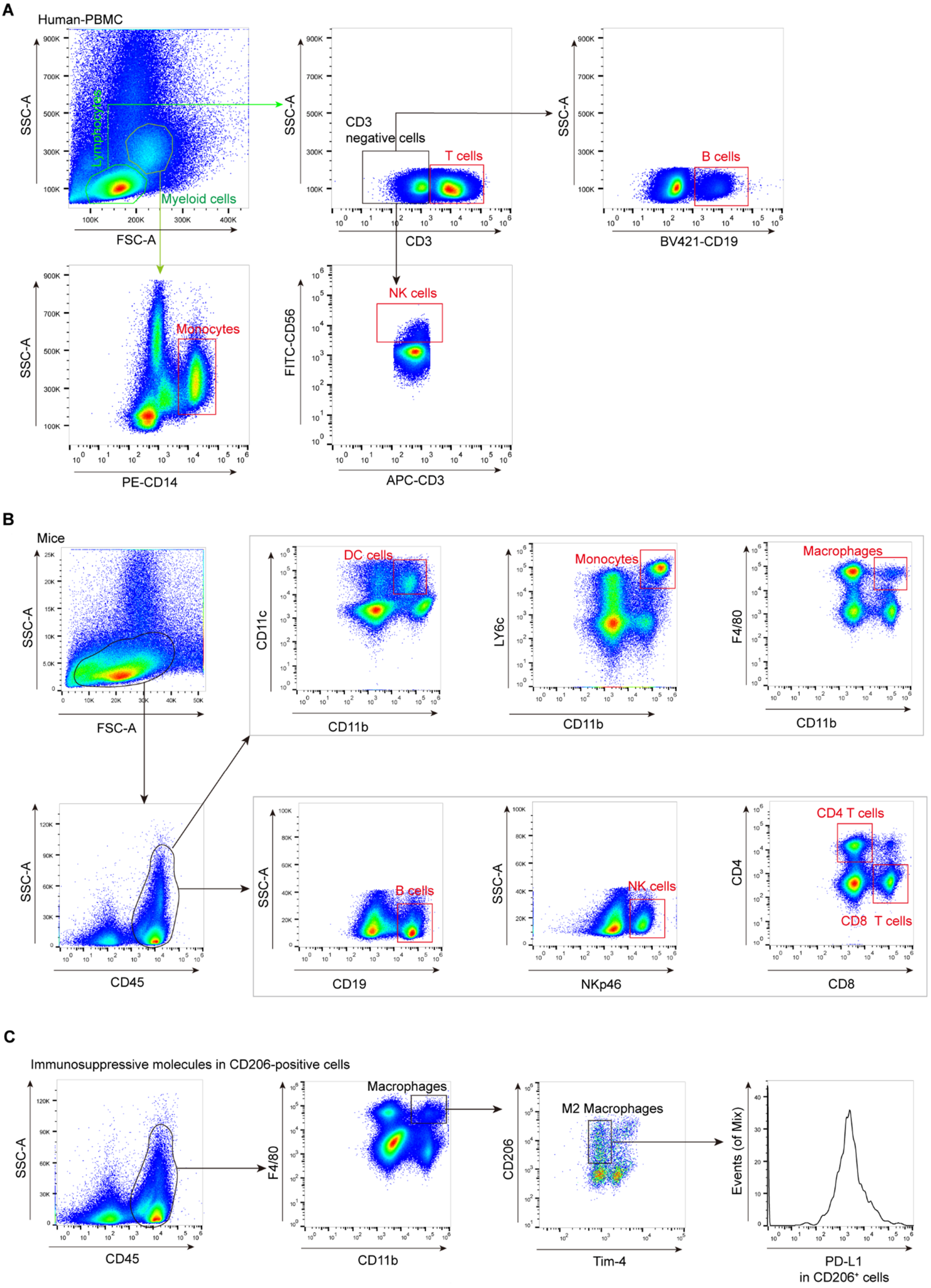
A
Human-PBMC
SSC-A
FSC-A
Lymphocytes
Myeloid cells
CD3
negative cells
T cells
CD3
B cells
BV421-CD19
NK cells
FITC-CD56
APC-CD3
Monocytes
PE-CD14
B
Mice
DC cells
CD11c
CD11b
Monocytes
LY6c
Macrophages
F4/80
CD45
B cells
CD19
NK cells
NKp46
CD4 T cells
CD8 T cells
CD4
CD8
C
Immunosuppressive molecules in CD206-positive cells
M2 Macrophages
CD206
Tim-4
Events (of Mix)
PD-L1
in CD206⁺ cells

Figure S14: Gating strategy used for flow cytometry.

A. Gating strategy for mononuclear cells isolated from peripheral blood.

B. Gating strategy for leukocytes and myeloid cells isolated from harvested tumor tissues.

C. Gating strategy for immunosuppressive macrophages.
