# Supplementary material for: Extracellular Vesicle‐Transferred ATP‐Citrate Lyase Induces Monocyte Differentiation Toward Tumor‐Associated Macrophages and Fuels Hepatocellular Carcinoma Progression
Source: Adv Sci (Weinh). 2026 Apr 17;13(35):e21458. doi: 10.1002/advs.202521458 (PMC13292156; doi:10.1002/advs.202521458)
Supplement: Supplementary file 2 — Supporting File 2: advs75345‐sup‐0002‐TableS1‐S6.pdf. [file ADVS-13-e21458-s001.pdf]

Supplementary Table 1. Cell lines used in this study

Cell line	Source	Identifier	Purchase Date	Contamination Free	Passage
SNU449	ATCC	Cat# CRL-2234; RRID: CVCL_0454	2020-06-05	Yes	P6-12
THP1	ATCC	Cat# TIB-202; RRID: CVCL_0006	2024-02-08	Yes	P3-7
HEK-293T	ATCC	Cat# CRL-11268 RRID: CVCL_0063	2023-03-03	Yes	P3-7
HHL5	BLUE FBIO	Cat# BFN6072012687 RRID: CVCL_S956	2020-06-05	Yes	P6-12
Hepa1-6	ATCC	Cat# CRL-1830 RRID: CVCL_0327	2020-06-05	Yes	P6-12
Raw 264.7	ATCC	Cat# TIB-71 RRID: CVCL_0493	2020-06-05	Yes	P3-7
HepG2	ATCC	Cat# HB-8065 RRID: CVCL_0027	2020-06-05	Yes	P6-12

Supplementary Table 2. Antibodies used in this study

Reagent	Source	Identifier
**Antibodies used in Western blot**		
Anti-ACLY antibody	Abcam	Cat# ab53007
Anti-TSG101 antibody	Abcam	Cat# ab133586
Anti-CD63 antibody	Abcam	Cat# ab271286
Anti-CD81 antibody	CST	Cat# 56039
Anti-PD-L1 antibody	Abcam	Cat# ab205921
Anti-H3K14Ac antibody	CST	Cat# 7627
Anti-H3K9Ac antibody	CST	Cat# 9649
Anti-H4K5Ac antibody	CST	Cat# 8647
Anti-H4K12Ac antibody	CST	Cat# 2591
Anti-H3 antibody	CST	Cat# 9715
Anti-H4 antibody	CST	Cat# 2592
Anti-GAPDH antibody	CST	Cat# 2118
Anti-beta actin antibody	CST	Cat# 3700
Anti-mouse HRP	CST	Cat# 7076V
Anti-rabbit HRP	CST	Cat# 7074V
**Antibodies used in immunohistochemistry and immunofluorescence**		
Anti-CD206	Abcam	Cat# ab64693
Anti-MERTK	Abcam	Cat# ab52968
Anti-MERTK	Invitrogen	Cat# 14-9053
Anti-CD276	Proteintech	Cat# 66481-1-Ig
Anti-Ki67	CST	Cat# ab15580
Anti-cleaved caspase 3	CST	Cat# 9661
Anti-CD68	ZSGB-BIO	Cat# ZM-0464

Anti-CD14	ZSGB-BIO	Cat# ZM-0532
Anti-CD81	Proteintech	Cat# 27855-1-AP
Anti-CD81	Proteintech	Cat# 66866-1-AP
Anti-ACLY	Proteintech	Cat# 15421-1-AP
Anti-ACLY	Abcam	Cat# ab40793
Anti-CD63	Proteintech	Cat# 25682-1-AP
Anti-CD56	Invitrogen	Cat# MA1-06801
Anti-PDL1	Proteintech	Cat# 66248-1-AP
Anti-CD8	Proteintech	Cat# 66868-1-AP
Anti-CD14	Proteintech	Cat# 17000-1-AP
Anti-rabbit IgG	Vector	Cat# MP-7401
Anti-mouse IgG	Vector	Cat# MP-7402
Anti-mouse IgG/AP+Anti-rabbit IgG/HRP	ZSGB-BIO	Cat# DS-0004
Anti-mouse IgG (H+L), Alexa Fluor ™ 555	Invitrogen	Cat# A-21422
Anti-rabbit IgG (H+L), Alexa Fluor™ 555	Invitrogen	Cat# A-31572
Anti-rabbit IgG (H+L), Alexa Fluor™ 488	Invitrogen	Cat# A-21206
**Antibodies used in flow cytometry**		
PE/Cy7 anti-mouse CD86	Biolegend	Cat# 105115
APC/Cy7 anti-mouse CD11b	Biolegend	Cat# 101225
FITC anti-mouse CD45	Biolegend	Cat# 103107
PE anti-mouse HLA-DR	Biolegend	Cat# 107607
APC anti-mouse F4/80	Biolegend	Cat# 123115

FITC anti-mouse CD206	Biolegend	Cat# 141703
Biotin anti-mouse CD206	Biolegend	Cat# 141713
PE anti-mouse PD-L1	Biolegend	Cat# 124307
APC anti-mouse CD276	Biolegend	Cat# 135607
PE/Cy7 anti-mouse MERTK	Biolegend	Cat# 151521
APC/Cy7 anti-mouse IL10	Biolegend	Cat# 505035
FITC anti-mouse CD8	Biolegend	Cat# 100705
AF700 anti-mouse CD3	Biolegend	Cat# 100215
PE anti-mouse CD56	Biolegend	Cat# 108707
Percp-Cy5.5 anti-mouse Granzyme B	Biolegend	Cat# 372211
PE/Cy7 anti-mouse CD4	Biolegend	Cat# 116015
APC/Cy7 anti-mouse CD45	Biolegend	Cat# 103115
PE/Cy7 anti-human CD206	Biolegend	Cat# 321123
Biotin anti-human CD206	Biolegend	Cat# 321118
PE anti-human PD-L1	Invitrogen	Cat# 12-5983-42
PE anti-human PD-L1	BD Biosciences	Cat# 557924
PE anti-Human CD274	BD Biosciences	Cat# 557924
Brilliant Violet 421™ anti-human CD4	Biolegend	Cat# 317434
eF450 anti-human CD4	Invitrogen	Cat# 48-0049-42
PE anti-human CD8	Beckman	Cat# IM0452U
PC7 anti-human CD8	Beckman	Cat# 6607102
FITC anti-human CD14	Beckman	Cat# IM0645U
FITC anti-human CD56	Invitrogen	Cat# 11-0569-42
FITC anti-human CD3	Beckman	Cat# IM2467

Brilliant Violet 421™ anti-human CD19	Biolegend	Cat# 302234
APC anti-mouse CD45R/B220	BD Biosciences	Cat# 553092
PE/Cy7 anti-mouse CD11b	BD Biosciences	Cat# 561098
AF700 anti-mouse/human CD11b	Biolegend	Cat# 101222
PE anti-mouse CD11c	Biolegend	Cat# 117308
PE/Cy7 anti-mouse CD19	Biolegend	Cat# 552854
AF700 anti-mouse CD3	BD Biosciences	Cat# 56-0032-82
PE/Cy7 anti-mouse CD4	BD Biosciences	Cat# 25-0041-81
FITC Rat Anti-Mouse CD45	BD Biosciences	Cat# 553079
Brilliant Violet 570™ anti-mouse CD45	Biolegend	Cat# 103136
PE anti-mouse CD69	Invitrogen	Cat# 12-0691-83
FITC anti-mouse CD86	Biolegend	Cat# 105005
APC anti-mouse F4/80	Biolegend	Cat# 123116
PE/Cy7 anti-mouse I-A/I-E	Biolegend	Cat# 107629
eF450 anti-mouse IFN-r	Invitrogen	Cat# 48-7311-82
PE anti-mouse IFN-γ	Biolegend	Cat# 505808
FITC anti-mouse IL10	BD Biosciences	Cat# 562037
FITC anti-mouse IL6	Invitrogen	Cat# 11-7061-81
PE anti-mouse NK1.1	Biolegend	Cat# 108707
PE/Cyanine7 anti-mouse PD-L1	Biolegend	Cat# 124314

Supplementary Table 3. Recognition sequences of shRNAs

shRNA	Recognition sequence (5' to 3')
Human ACLY shRNA1	CGTGAGAGCAATTCGAGATTA
Human ACLY shRNA2	GCCTCAAGATACTATACATTT
Mouse ACLY shRNA1	GCAGCAAAGATGTTCAGTAAA
Mouse ACLY shRNA2	GAAGGCAAGATCCTCATCATT
Human TKT shRNA1	TCACCGTGGAGGACCATTATT
Human TKT shRNA2	TAGAAGATCTGGCTATGTTTC
Human CAD shRNA1	CCCAGATGAAATGGATGAGTT
Human CAD shRNA2	TCGGGCTCTCAGGCAATTAAG
Human CD276 shRNA1	CAACGAGCAGGGCTTGTTTGA
Human CD276 shRNA2	TGCTGGAGAAAGATCAAACAG

Supplementary Table 4. Sequences of primers used for qPCR

Gene	Primer	Sequence (5' to 3')
GAPDH	Forward	GGAGCGAGATCCCTCCAAAAT
	Reverse	GGCTGTTGTCATACTTCTCATGG
18S	Forward	CTACCACATCCAAGGAAGCA
	Reverse	TTTTTCGTCACTACCTCCCCG
IL10	Forward	GACTTTAAGGGTTACCTGGGTTG
	Reverse	CACATGCGCCTTGATGTCTG
PD-L1	Forward	TGCAGGGCATTCCAGAAAGAT
	Reverse	CCGTGACAGTAAATGCGTTCAG
CD276	Forward	AGGGCAGCCTATGACATTCC
	Reverse	GGTCCTCAGCTCCTGCATTC

CD206	Forward	GTGATGGGACCCCTGTAACG
	Reverse	CTGCCCAGTACCCATCCTTG
MERTK	Forward	CGCTCTGGCGTAGAGCTATC
	Reverse	AGGCTGGGTTGGTGAAAACA
HLA-DRA	Forward	TGGCGGCTTGAAGAATTTGG
	Reverse	GGCTCTCTCAGTTCCACAGG
CD163	Forward	AGTCTGCTCAAGATACACAGAAA
	Reverse	GGTAGAAAGGGCAACTCCACA
NOS2	Forward	ATCTGCAGACACGTGCGTTA
	Reverse	GTCGATGCACAGCTGAGTGA
ACLY	Forward	GACTTCGGCAGAGACAGGTAG
	Reverse	AGGAGTTCTTTGCCCGTCTG
IL1B	Forward	ATGATGGCTTATTACAGTGGCAA
	Reverse	GTCGGAGATTCGTAGCTGGA
IL6	Forward	ACTCACCTCTTCAGAACGAATTG
	Reverse	CCATCTTTGGAAGGTTCAGGTTG

Supplementary Table 5. Immune inhibitory genes

FOXP3	LAIR1	LILRB2	SIGLEC15	CD244
PTGS2	CD33	LAG3	TGFB2	CD200
TNFSF14	TNFRSF14	PILRA	LILRA1	PDCD1
S100A9	CD47	SIGLEC5	C10orf54	CD300LB
LILRB3	CLEC12A	IL10	SELPLG	MERTK
S100A8	HAVCR2	LILRB1	FCGR2B	TIGIT
CLEC4A	PDCD1LG2	FCER2	CD274	PVR
IGF2	CD300LF	NT5E	CTLA4	LILRA5
ARG1	SIRPA	SIGLEC9	TNFRSF18	CD276

Supplementary Table 6. Clinical information of a local HCC cohort

HCC cohort	Maximum Tumor Diameter (cm)	Tumor Grade	Overall Patient Survival (month)
Patient 1	12.8	T4	2.9
Patient 2	1.2	T1a	30.1
Patient 3	2	T1a	37.7
Patient 4	2	T1a	45.2
Patient 5	3.5	T1b	39.7
Patient 6	3	T1b	40.9
Patient 7	3	T1b	47.7
Patient 8	5	T1b	65.8
Patient 9	8.5	T1b	67.2
Patient 10	4	T1b	74.0
Patient 11	5.5	T2	5.3
Patient 12	8.6	T2	3.06
Patient 13	3	T2	8.6
Patient 14	11.5	T4	11.4
Patient 15	5	T2	36.3
Patient 16	2.5	T2	38.2
Patient 17	5	T2	40.9
Patient 18	3	T2	49.8
Patient 19	2	T2	52.5
Patient 20	10	T3	24.6
Patient 21	7	T3	71.6
Patient 22	11.5	T4	11.4
Patient 23	8	T3	76.6
